# Prediction of Drug-Drug Interactions Arising from CYP3A induction Using a Physiologically Based Dynamic Model[Fn FN2]

**DOI:** 10.1124/dmd.115.066845

**Published:** 2016-06

**Authors:** Lisa M. Almond, Sophie Mukadam, Iain Gardner, Krystle Okialda, Susan Wong, Oliver Hatley, Suzanne Tay, Karen Rowland-Yeo, Masoud Jamei, Amin Rostami-Hodjegan, Jane R. Kenny

**Affiliations:** Simcyp (a Certara Company), Sheffield, United Kingdom (L.M.A., I.G., O.H., K.R.-Y., M.J., A.R.-H.); DMPK, Genentech Inc., South San Francisco, California (S.M., K.O., S.W., S.T., J.R.K.); and Manchester Pharmacy School, University of Manchester, United Kingdom (A.R.-H.)

## Abstract

Using physiologically based pharmacokinetic modeling, we predicted the magnitude of drug-drug interactions (DDIs) for studies with rifampicin and seven CYP3A4 probe substrates administered i.v. (10 studies) or orally (19 studies). The results showed a tendency to underpredict the DDI magnitude when the victim drug was administered orally. Possible sources of inaccuracy were investigated systematically to determine the most appropriate model refinement. When the maximal fold induction (*Ind*_max_) for rifampicin was increased (from 8 to 16) in both the liver and the gut, or when the *Ind*_max_ was increased in the gut but not in liver, there was a decrease in bias and increased precision compared with the base model (*Ind*_max_ = 8) [geometric mean fold error (GMFE) 2.12 vs. 1.48 and 1.77, respectively]. Induction parameters (mRNA and activity), determined for rifampicin, carbamazepine, phenytoin, and phenobarbital in hepatocytes from four donors, were then used to evaluate use of the refined rifampicin model for calibration. Calibration of mRNA and activity data for other inducers using the refined rifampicin model led to more accurate DDI predictions compared with the initial model (activity GMFE 1.49 vs. 1.68; mRNA GMFE 1.35 vs. 1.46), suggesting that robust in vivo reference values can be used to overcome interdonor and laboratory-to-laboratory variability. Use of uncalibrated data also performed well (GMFE 1.39 and 1.44 for activity and mRNA). As a result of experimental variability (i.e., in donors and protocols), it is prudent to fully characterize in vitro induction with prototypical inducers to give an understanding of how that particular system extrapolates to the in vivo situation when using an uncalibrated approach.

## Introduction

Over recent years, the use of in vitro-in vivo extrapolation linked with physiologically based pharmacokinetic (IVIVE-PBPK) models that integrate key in vitro drug parameters with human system parameters (e.g., demography, physiology, genetics) to predict pharmacokinetics and drug-drug interactions (DDIs) and to assist in decision making has become increasingly common (EMA, 2012; Rostami-Hodjegan et al., 2012; Huang et al., 2013). More recently, these approaches have also been used to inform the wording of product information labels (Janssen Biotech, 2013a,b; Imbruvica: Highlights of Prescribing Information, http://www.imbruvica.com/downloads/Prescribing_Information.pdf’ and Olysio: Highlights of Prescribing Information, http://www.olysio.com/shared/product/olysio/prescribing-information.pdf). In particular, the benefits of adopting mechanistic approaches (including information on both the perpetrator and victim drug, e.g., fraction metabolized (*fm*) and fraction metabolized in the gut (*F*_G_) over purely pragmatic approaches have been recognized (Einolf, 2007; Almond et al., 2009; FDA, 2012). Mechanistic models can be further classified as either dynamic or static. *Static models* assume a constant perpetrator concentration throughout the full dosing interval and ignore temporal changes in concentrations, whereas *dynamic models* account for changes in perpetrator concentration with time (Einolf, 2007; Almond et al., 2009; EMA, 2012; FDA, 2012). The concentration used as the input (driving) concentration for the prediction drug interactions [e.g., inlet (portal vein) vs. outlet (liver) vs. *C*_max_ (systemic)] and whether the total or unbound concentrations that are used can vary across static methods (Almond et al., 2009), with some regulatory guidance favoring more cautious approaches using total concentrations in the basic models but unbound concentrations in the mechanistic static models (FDA, 2012). Although the overall effect of time-dependent inhibition and induction at the new enzyme steady-state level can be simulated only by using static approaches, investigation of the time course can be simulated using dynamic models that factor in the changing concentrations of substrate, perpetrator, as well as enzyme. An additional advantage of the dynamic models (particularly in the case of competitive inhibition) is to enable evaluation of the dosing schedule dependence of the DDI and possible strategies to minimize such effects.

Although dynamic approaches have increased complexity compared with static approaches, they make fewer assumptions and are necessary if the intention is to account for phenomena such as autoinduction, where the perpetrator induces enzyme levels, in turn increasing its own metabolism and thereby altering concentrations achieved with subsequent doses. This in turn impacts the level of enzyme achieved when the system reaches steady state. Here, our focus is the dynamic prediction of induction potential of a new drug using IVIVE-PBPK, as implemented in the Simcyp simulator (Almond et al., 2009), where in vitro data for a new drug is calibrated against in vitro data for a compound with known induction potential as a positive control (e.g., rifampicin). The effect of the unknown drug in vivo can then be predicted based on the difference in potency of the new compound compared with rifampicin and the plasma levels achieved after dosing in vivo in humans.

Numerous independent publications have described the dynamic induction model within the Simcyp simulator as being successfully applied for the quantitative prediction of CYP3A4 induction (Gandelman et al., 2011; Xu et al., 2011; Dhuria et al., 2013; Greupink et al., 2013; Einolf et al., 2014); however, we have noted cases of under prediction in the interaction between rifampicin and orally dosed midazolam (MDZ). The success of IVIVE approaches to predict enzyme induction depends on a number of factors, including the type (induction of mRNA vs. enzyme activity) and quality of in vitro data, the methods used to analyze the in vitro data, the approach taken to scale the in vitro data to the in vivo situation (use of calibrators for in vitro and in vivo induction data), as well as variability in the data from the clinical studies against which the predictions are compared. In this study, a systematic evaluation of an IVIVE-PBPK approach to predict the interactions between rifampicin and CYP3A substrates with ranging *fm*_3A4_ (the fractional contribution of CYP3A4 to systemic clearance) and F_G_ (the fraction escaping gut wall metabolism) was carried out. Model refinements to improve the prediction accuracy were investigated and then applied to predict the interaction with other independent CYP3A inducers, using rich in vitro data generated using multiple human hepatocyte donors within a single laboratory and standardized protocols.

## Materials and Methods

### 

#### Materials.

Cryopreserved human hepatocytes from four donors (Hu1206, Hu1191, Hu1198, Hu4193), cryopreserved hepatocytes recovery media, and AlamarBlue cell viability reagent were purchased from Life Technologies (Grand Island, NY). InvitroGro culture media (CP and HI) and Torpedo antibiotic mix were purchased from BioreclamationIVT (Baltimore, MD). QuantiGene Plex 2.0 assay kits (panel no. 11477) were purchased from Affymetrix (Santa Clara, CA). Dimethyl sulfoxide, rifampicin, testosterone, phenobarbital (PHB), carbamazepine (CBZ), and phenytoin (PHY) were purchased from Sigma-Aldrich (St. Louis, MO).

#### Generation of Induction Parameters In Vitro.

The changes in mRNA and enzyme activity were assessed in parallel in cryopreserved human hepatocytes from four donors using previously described methods (Halladay et al., 2012). In brief, hepatocytes were incubated with varying concentrations of prototypical inducers (serial dilutions of inducers in dimethyl sulfoxide were prepared daily) before the assessment of activity (measurement of 6*β*-hydroxytestosterone formation measured by liquid chromatography-tandem mass spectroscopy (LC-MS/MS)] and mRNA levels (QuantiGene Plex 2.0 Affymettrix assay kit). Cell toxicity and cell viability were monitored using lactate dehydrogenase leakage and AlamarBlue assays (Halladay et al., 2012). The concentration ranges (Table 1) were selected for each inducer based on previous published studies with the aim of determining a robust *Ind*_max_ and *IndC*_50_.

**TABLE 1:** Final concentrations of inducer in culture medium with 0.1% dimethyl sulfoxide (v/v)

Inducer	Concentrations
	*µ*M
Rifampicin	0.03, 0.1, 0.3, 1, 3, 10, 30
Carbamazepine	1, 3, 10, 30, 100, 300, 1000
Phenytoin	1, 3, 10, 30, 100, 300, 1000
Phenobarbital	10, 30, 100, 300, 1000, 2000, 3000
Efavirenz	0.1, 0.3, 1, 2, 3, 10, 30
Nifedipine	0.03, 1, 2, 3, 10, 30, 100

#### In Vitro Data Analysis.

Data for mRNA and activity were plotted as fold increase over vehicle control versus the concentration of the inducer. Curve fitting was carried out on data from each hepatocyte donor individually and then mean *Ind*_max_ (maximum fold induction, *E*_max_ + 1) and *IndC*_50_ (the concentration that yields half of the E_max_) were calculated. Both three-parameter (assuming the Hill exponent is equal to 1) and four-parameter sigmoidal models were fitted to the in vitro data (mRNA and activity) using GraphPad Prism (version 5). Parameters derived from these two models were not significantly different; therefore, the values from the simpler model (three-parameter fit) were used for subsequent analysis (Table 2). It should be noted that *Ind*_max_ is the maximum fold induction and as such is not corrected for baseline (i.e., is equal to *E*_max_ + 1). Values are entered as *Ind*_max_, and this correction is handled within the Simcyp Simulator (see eq. 3 and eq. 4).

**TABLE 2:** In vitro induction parameters (*Ind*_max_ and *IndC*_50_) for rifampicin, carbamazepine, phenobarbital, and phenytoin generated using mRNA and activity data Data are shown as the mean and standard deviation from four human hepatocyte donors. Where Ind_max_ is the maximum fold induction (equal to *E*_max_ +1) and *IndC*_50_ is the concentration that gives half maximal fold induction (analogous to *EC*_50_).

		Activity		mRNA	
		*Ind*_max_	*IndC*_50_	*Ind*_max_	*IndC*_50_
		* fold*	*µM*	*fold*	*µM*
Rifampicin	Mean	22.7	0.30	29.9	0.71
	S.D.	7.8	0.10	7.0	0.35
Carbamazepine	Mean	16.6	59.1	21.9	58.7
	S.D.	6.1	37.3	12.4	18.0
Phenobarbital	Mean	21.1	473	44.2	743
	S.D.	11.5	245	25.9	334
Phenytoin	Mean	13.6	51.3	24.5	123
	S.D.	3.7	29.4	7.6	120
Efavirenz	Mean	13.5	4.9	18.1	8.4
	S.D.	4.2	1.7	5.4	5.1
Nifedipine	Mean	15.6	4.0	30.0	13.0
	S.D.	11.3	1.9	22.0	9.5

#### Clinical Pharmacokinetic Data for the Assessment of Prediction Accuracy.

PubMed and The Metabolism & Transport Drug Interaction Database (http://www.druginteractioninfo.org/applications/metabolism-transport-drug-interaction-database/) were used to identify relevant clinical DDI data arising from induction in white subjects. DDI studies involving the CYP3A4 inducer rifampicin with the CYP3A4 substrates MDZ, alfentanil, alprazolam, nifedipine, simvastatin, and zolpidem were identified. In vivo studies were included in the analysis if the report included sufficient details of the dosage regimen to allow accurate replication of the trial design as well as the fold-change in the plasma area under the curve (AUC). Where concentration-time profiles were available in the references, these data were digitized (GetData software http://getdata-graph-digitizer.com/index.php) and compared with the predicted concentration-time profiles.

Fifteen clinical studies describing the disposition of MDZ, before and after multiple dosing with rifampicin, were identified. Of these studies, one study was excluded because the data were from subjects of mixed ethnicity, only one-third of whom were white (Adams et al., 2005), and the data were not stratified in a way that allowed simulation of the different ethnic groups independently. Similarly, data from the i.v. MDZ arm from the study by Floyd et al. (2003) could not be used, although data from female white subjects after an oral dose were described and hence were included (Floyd et al., 2003). In the study by Eap et al. (2004), CYP3A4 induction was assessed with 7.5 and 0.075 mg of orally administered MDZ on consecutive days. The magnitude of interaction with the 0.075-mg dose was much lower than for the 7.5-mg dose (AUC ratio 2.3- vs. 19.1-fold), which may be due to issues with the limit of detection after induction of CYP3A, and so only the 7.5-mg data from this study have been included. All other studies were included to assess the prediction accuracy of the model. Information describing the dosing regimen, the route of administration of MDZ, and the study size is provided for the remaining studies in Table 3.

**TABLE 3:** Rifampicin-mediated drug-drug interaction studies reported in the literature Details of the exposure of CYP3A4 probe substrate in the before and after multiple dosing of rifampicin are shown. A negative dose stagger indicates that the victim was dosed before the perpetrator. Data are expressed as mean (coefficient of variation) with the exception of those given.

Study	Rifampicin	Victim (Dose)	Dose Stagger	*n*	AUC	AUCi	1/AUC Ratio
i.v. administration of victim drugs					ng/mL.h	ng/mL.h	
Link et al., 2008	600 mg daily for 6 days	MDZ (2 mg)	24	8	126 (84–269)^*a*^	82.4 (58.8–102)*^a^*	1.53
Kharasch et al., 2004	600 mg daily for 5 days	MDZ (1 mg)	12*^c^*	10	28.4 (14.1)	14.8 (18.2)	1.92
Gorski et al., 2003	600 mg daily for 7 days	MDZ (0.05 mg/kg)	12	52	118 (35.4)	52.8 (29.7)	2.23
Phimmasone and Kharasch, 2001	600 mg daily for 5 days	MDZ (1 mg)	12	6	53.0 (26.4)	25.5 (19.0)	2.08
Szalat et al., 2007*^h^*	600 mg daily for 7 days	MDZ (0.05 mg/kg)	12*^c^*	3	89.5 (18.3)	51.8 (13.5)	1.73
Kharasch et al., 1997	600 mg daily for 5 days	MDZ (1 mg)	24	9	72.2*^d^* (n/a)	27.4d (n/a)	2.64
Holtbecker et al., 1996	600 mg daily for 7 days	NIF (0.02 mg/kg)	0	6	38.1 (12.6)	26.7 (44.9)	1.43
Phimmasone and Kharasch, 2001	600 mg daily for 5 days	ALF (0.015 mg/kg))	13*^c^*	6	111 (52.1)	48.2 (19.7)	2.31
Kharasch et al., 2004	600 mg daily for 5 days	ALF (0.015 mg/kg)	13*^c^*	10	64.8 (41.0)	24.3 (26.7)	2.67
Kharasch et al., 2011	600 mg daily for 6 days	ALF (1 mg)	9*^c^*	6	59.0 (45.8)	21.0 (38.1)	2.81
Oral administration of victim drugs							
Backman et al., 1996	600 mg daily for 5 days	MDZ (15 mg)	17	10	170 (23.4)	7.00 (40.6)	24.3
Backman et al., 1998	600 mg daily for 5 days	MDZ (15 mg)	17	9	277 (78.0)	4.40 (68.2)	63.0
Chung et al., 2006	600 mg daily for 9 days	MDZ (0.075 mg/kg)	−2	18	49.0 (22–103)*^b^*	6.10 (125–371)*^b^*	8.03
Eap et al., 2004	450 mg daily for 5 days	MDZ (7.5 mg)	12*^c^*	4	67.0 (44.8)	3.50 (5.70)	19.1
Gurley et al., 2006	300 mg twice a day for 7 days	MDZ (8 mg)	0*^c^*	19	79.6 (29.1)	4.55 (49.2)	17.5
Gurley et al., 2008	300 mg twice a day for 7 days	MDZ (8 mg)	2	16	107 (38.0)	6.46 (54.3)	16.6
Link et al., 2008	600 mg daily for 6 days	MDZ (7.5mg)	24	8	103 (64–164)*^a^*	1.60 (1–7.2)*^a^*	64.3
Reitman et al., 2011	600 mg daily for 28 days	MDZ (2 mg)	0	11	21.4 (33.6)	2.64 (45.3)	8.11
Kharasch et al., 2004	600 mg daily for 6 days	MDZ (3 mg)	12*^c^*	10	20.9 (20.1)	1.10 (45.5)	19.0
Floyd et al., 2003	600 mg daily for 16 days	MDZ (2 mg; 25 mg)*^e^*	0	12*^g^*	27.1 (n/a)	19.9 (n/a)	17.0*^f^*
Gorski et al., 2003	600 mg daily for 7 days	MDZ (4 mg; 6 mg)*^e^*	12	52	35.8 (58.1)	3.70 (75.7)	25.6*^f^*
Schmider et al., 1999	450 mg daily for 4 days	APZ (1 mg)	0*^c^*	4	242 (31.3)	28.4 (23.9)	8.53
Chung et al., 2006	600 mg daily for 9 days	SMV (40 mg)	−2	18	29.0 (8–56)*^b^*	2.60 (0.8–26)*^b^*	11.2
Kyrklund et al., 2000	600 mg daily for 28 days	SMV (40 mg)	0	10	17.3 (57.2)	2.40 (75.4)	7.21
Holtbecker et al., 1996	600 mg daily for 7 days	NIF (20 mg)	0	6	230 (14.7)	18.8 (45.7)	12.2
Villikka et al., 1997b	600 mg daily for 5 days	ZOL (20 mg)	17	8	1110 (36.9)	332 (56.4)	3.34
Kharasch et al., 2004	600 mg daily for 6 days	ALF (0.06 mg/kg)	13*^c^*	10	103 (29.1)	4.70 (97.9)	21.9
Kharasch et al., 2011	600 mg daily for 5 days	ALF (4 mg)	12*^c^*	6	108 (63.0)	6.40 (50.0)	16.9
Villikka et al., 1997a	600 mg daily for 5 days	TZM (0.5 mg)	17	10	14.8 (21.4)	0.74 (59.8)	20.0

ALF, alfentanil; APZ, alprazolam; AUC, area under the curve; MDZ, midazolam; n/a, not available; NIF, nifedipine; ROA, root of administration; SMV, simvastatin; TZM, triazolam; ZOL, zolpidem.

^*a*^ Median and range.

^*b*^ Geometric mean and range.

^*c*^ Ambiguous.

^*d*^ Calculated assuming a body weight of 70 kg in both control and rifampicin arms of the study.

^*e*^ Dose escalated for the RIF arm of the study to give equivalent MDZ concentrations as at baseline;

^*f*^ The ratio of clearance due to dose escalation.

^*g*^ Midazolam AUC in the absence and presence of rifampicin were calculated from oral clearances provided for 12 white subjects (all women) of the 57 subjects studied in total. Data for white men were not provided.

^*h*^ Cerebrotendinous xanthomatosis (CTX) patients.

As none of the DDI studies identified above described the concentration-time profiles of rifampicin, independent studies were identified for the performance verification of rifampicin exposure. Of these, two studies were carried out in white healthy volunteers (Acocella et al., 1971; Drusano et al., 1986) and were used to evaluate the simulated concentration-time profiles of rifampicin.

Further literature searching was carried out to identify DDI investigations of other inducers (CBZ, PHY, and PHB) with the CYP3A substrates. A total of six studies were identified as summarized in Table 4.

**TABLE 4:** Summary of the clinical drug-drug interactions studies available within the literature The exposure of CYP3A4 probe substrate before and after multiple dosing of carbamazepine, phenytoin, and phenobarbital are shown Data are expressed as mean (coefficient of variation) with the exception of those where the individual data are provided (n = 2).

Study	Inducer	Victim (Dose)	Dose Stagger	*n*	AUC (mg/L.h)	AUCi (mg/L.h)	1/AUC Ratio
Carbamazepine							
Ucar et al., 2004	CBZ (200 mg daily for 2 days; 300 mg twice a day for 12 days)	SMV (80 mg)	0	12	0.089 (58.1)	0.023 (56.7)	3.93
Andreasen et al., 2007	CBZ (200 mg twice a day for 2 days; 400 mg twice a day for 14 days)	QND (200 mg)	0*^a^*	10	5.12 (n/a)	1.98 (n/a)	2.57
Vlase et al., 2011	CBZ (400 mg daily for 16 days)	ZOL (5 mg)	0^a^	18	0.235 (70.4)	0.102 (58.1)	2.31
Phenytoin							
Data et al., 1976	Dose adjusted to maintain plasma PHY conc 10–20 *μ*g/ml	QND (300 mg)	0*^a^*	2	12.6 (10.3, 15.0)	5.53 (4.24, 6.82)	2.28
Phenobarbital							
Schellens et al., 1989	PHB (100 mg daily for 8 days)	NIF (20 mg)	12*^a^*	15	0.343 (36.4)	0.135 (57.8)	2.54
Data et al., 1976	Dose adjusted to maintain plasma PHB conc. 10–20 *μ*g/ml	QND (300 mg)	0^a^	2	12.0 (9.33, 14.6)	4.10 (3.19, 5.00)	2.92

AUC, area under the curve; CBZ, carbamazepine; n/a, not available; PHB, phenobarbital; PHY, phenytoin; QND, quinidine; SMV, simvastatin; ZOL, zolpidem.

^*a*^ Ambiguous.

#### PBPK Modeling.

Populations of virtual human subjects were generated in the Simcyp Population-based Simulator using a correlated Monte Carlo approach (Jamei et al., 2009). A minimal (or lumped) PBPK model of distribution was assumed for all compounds, where all organs other than the intestine and liver are combined (Rowland Yeo et al., 2010).

With the exception of data describing the induction efficacy and potency, in vitro and pharmacokinetic data for substrates (Supplemental Table 1) and inducers (Supplemental Table 2) were taken from the literature. In cases where data were available from more than one independent source for the same parameter, they were combined to give weighted means based on the number of observations. With the exception of alfentanil and PHB, the compound files were taken from those released in version 12 Release 2 of the Simcyp simulator with any subsequent updates highlighted (Supplemental Material).

For each of the CYP3A substrates used in this study and for two of the four perpetrators (CBZ and PHY) sufficient in vitro metabolism information was available to simulate the contribution of different enzymes to the overall elimination of the compound. These data were used as input data to the Simcyp simulator and extrapolated to predict the intrinsic clearance in the whole liver and gut in both the absence and presence of an inducer. For the other compounds (rifampicin and PHB) assessed as drug-interaction perpetrators, CL was defined from in vivo estimates of systemic and oral clearance, respectively. The PBPK model was then used to simulate the time course of victim, perpetrator, and levels of the active CYP3A4 enzyme (in the liver and gut) of each virtual subject. The effect of autoinduction was automatically considered where the metabolism of the inducer is adequately defined (e.g., for CBZ and PHY).

The differential equations describing the kinetics of victim and perpetrator drugs and enzyme dynamics for inhibition have been reported in full previously (Rowland Yeo et al., 2010). Here, we focus only on the equations describing time variant intrinsic clearance of the victim in the presence of a perpetrator compound in the liver and gut (eq. 1 and eq. 2). The effect of competitive inhibition between substrate and perpetrator is described by the terms *I*_Liv_, *I*_pv_, and *K*_iu-e_, effects due to enzyme induction or mechanism based inhibition are incorporated by time-dependent changes in the levels of active enzyme (ENZ_act,h_) (eq. 3 and eq. 4). Finally, the time-dependent value of intrinsic clearance is used in the differential equations used to calculate the plasma concentration time profile and AUC (Rowland Yeo et al., 2010):(1)
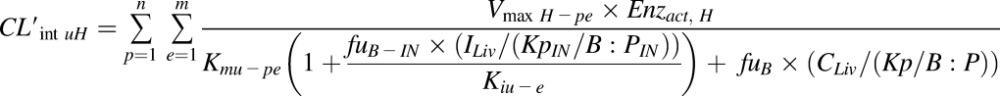
(2)
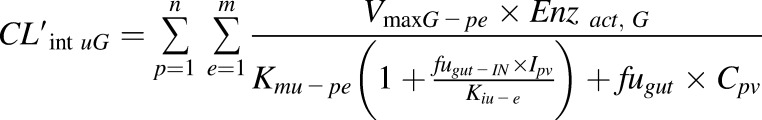
Where 
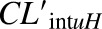
 and 
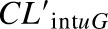
 are the unbound intrinsic clearance of substrate per whole liver and gut, respectively, in the presence of a perpetrator compound. 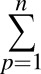
and 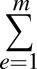
refer to the total number of pathways and enzymes involved in metabolism of the substrate, respectively. B:P and B:P_IN_ are the blood to plasma ratios of substrate and perpetrator 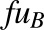 and 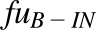 are the unbound fraction in plasma to the blood to plasma ratio (*fu* / B:P) of the substrate and perpetrator, respectively. *fu*_gut-IN_ is the fraction unbound in the gut. 
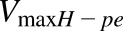
 and 
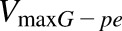
 are the maximum metabolic reaction velocity of substrate (victim) per whole liver and gut, respectively, 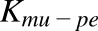 is the Michaelis constant (corrected for nonspecific binding); Enz_act,H_ and Enz_act,G_ is the amount of active enzyme, in this case CYP3A at any given time in the liver and gut, respectively; and 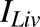 and 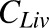 are the time varying liver concentrations of inhibitor and substrate, respectively, *K*p and *K*p_IN_ are the tissue to plasma partition coefficients of substrate and perpetrator. For compounds that show no competitive inhibition, the inhibition terms 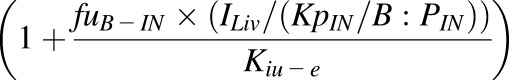 and 
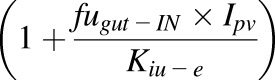
 for the liver and gut, respectively, equal to one and hence no inhibition is simulated:(3)
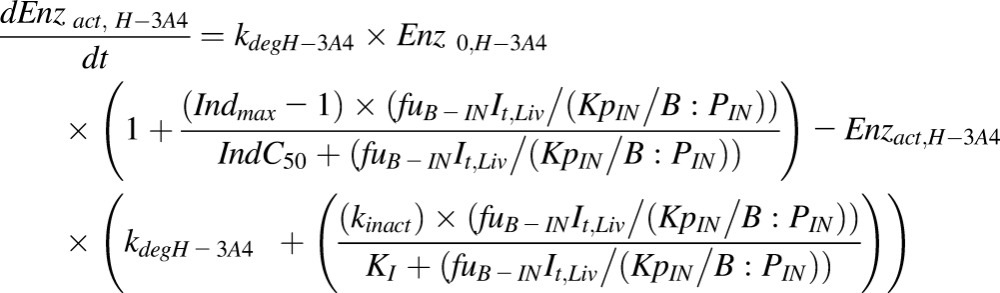
(4)
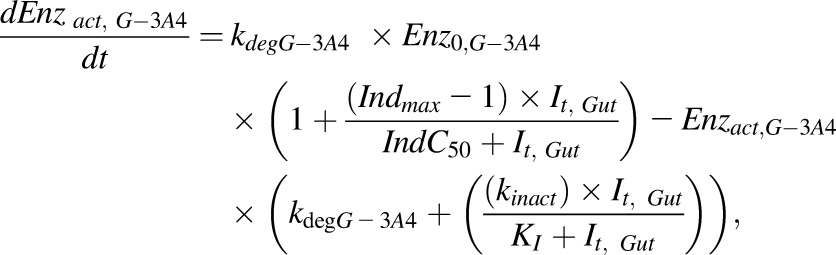
where 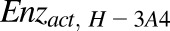 and 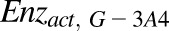 are the amounts of active CYP3A4 at a given time in the liver (eq. 3) and gut (eq. 4), respectively, *Enz*_0,H-3A4_ and Enz_0,G-3A4_ is the basal amount of CYP3A in the liver and gut, respectively, and (*Enz*_act_(t) = E_0_ at *t* = 0). *Ind*_max_ is the maximal fold induction expressed as a fold over vehicle control. *Ind*_max_ = *E*_max_ + 1. *Ind*C_50_ is the concentration that supports half-maximal induction; *K*_I_ is the concentration of mechanism-based inhibitor associated with half-maximal inactivation rate of the enzyme (*k*_inact_(1/h)); *I*_t_ is the perpetrator concentration at time *t* in either the liver or the gut.

#### Derivation of Reference In Vivo Induction Parameters and their Role in Calibration.

In vivo reference values describing the concentration–induction response of rifampicin (*Ind*_max_ and *IndC*_50_) were derived using a study describing the change in metabolic ratio of 6*β*-hydroxycortisol to cortisol following multiple dosing of rifampicin (600 mg daily 14 days) (Tran et al., 1999) in conjunction with concentration-time profile data (Acocella et al., 1971). These in vivo values for rifampicin are then used to calibrate the in vitro Ind_max_ and IndC_50_ values of other inducers/test compounds against in vitro values of rifampicin from the same experiment as shown in eq. 5 and eq. 6: (5)
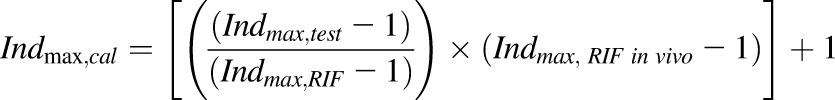
(6)
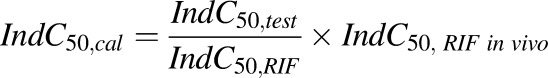
where *cal*, *test*, *RIF*, and *RIF* in vivo indicate whether the induction parameters are calibrated, the in vitro values of the test compound in a given assay, the in vitro values for rifampicin in a given assay and the reference in vivo values for rifampicin, respectively.

#### Design of Virtual Studies.

To ensure that the characteristics of virtual subjects reflected those of the subjects studied in vivo, the age range, proportion of males and females, and the number of subjects were matched to the information on individual clinical trials presented in the publications. The simulations were also matched to each published study in terms of dose, as well as the time, frequency, duration, and route of dosing for both the perpetrator (in this case an inducer of CYP3A4) and victim (a substrate of CYP3A4). For each simulation, 10 separate trials were generated to assess variability across groups. Although some of the victim drugs are metabolized by CYP3A5 in addition to CYP3A4, only CYP3A4 was considered as CYP3A5 induction is less well characterized and generally accepted as less significant compared with CYP3A4 (Williamson et al., 2011).

The accuracy of simulations that were run using in vivo reference values (*Ind*_max_ = 8; *IndC*_50_ = 0.32) for rifampicin itself and for calibration of other inducers was assessed (model A). The simulated plasma rifampicin concentrations and the simulated *fm*_3A4_ and *F*_G_ for the CYP3A4 substrates were verified against observed data. Parameters with uncertainty were identified, and sensitivity analysis was then used to assess which parameters were most likely to contribute to misprediction. Based on these analyses, simulations were repeated using different assumptions regarding the *Ind*_max_ and *IndC*_50_ values entered into the model as follows:

- Use of a higher *Ind*_max_ in the gut (16) than in the liver (8) but the same *IndC*_50_ in both sites of interaction (0.32) (model B)
- Use of a higher *Ind*_max_ in both the gut and liver (16) but the same *IndC*_50_ (0.32) (model C)
- Use of *Ind*_max_ and *IndC*_50_ values derived from in vitro data without calibration (mRNA) (model D)
- Use of *Ind*_max_ and *IndC*_50_ values derived from in vitro data without calibration (activity) (model E)
- Use of a higher *Ind*_max_ in both the gut and liver (12) but the same IndC_50_ (0.32) (model F)
- Use of a higher *Ind*_max_ in both the gut and liver (20) but the same IndC_50_ (0.32) (model G)

**Fig. 1.**
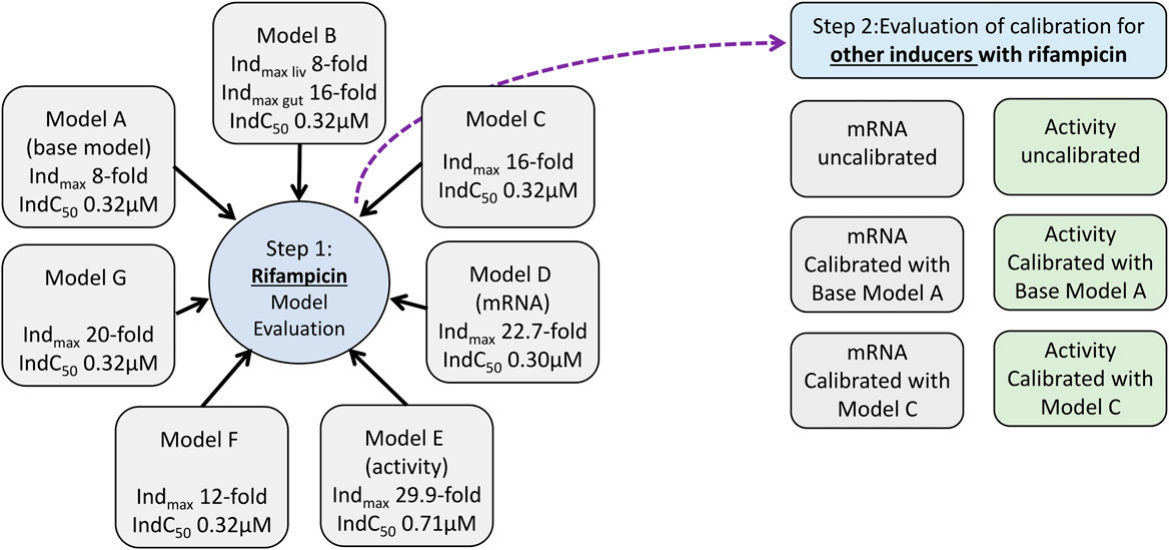
A schematic representation of the investigation that was split into two main stages: step 1 was the evaluation of different rifampicin models before the best model (model C) was evaluated for calibration of mRNA and activity data for the other inducers. This result was compared with no calibration and calibration with the original base model (model A).

After the best model was selected, the refined value of *Ind*_max_ was used to calibrate the in vitro data of the other inducers and the overall prediction accuracy for these inducers assessed. A schematic representation of this investigation is shown in Fig. 1.

#### Assessment of Prediction Accuracy.

The ratio of the AUC of the substrate in the absence and the presence of an inhibitor of substrate metabolism (AUC_(0–∞),inhibitor_/AUC_(0–∞),control_) and the percent of change in the AUC are commonly used as a basis for prediction of metabolic DDIs. In the presence of an enzyme inducer, this ratio gives values < 1; to aid interpretation, in this manuscript the reciprocal of this ratio has been used (AUC_(0-∞),control_ /AUC_(0-∞),induced_) to yield ratios > 1 in the presence of an enzyme inducer. However, data were plotted both ways to show the comparison. The means of AUC ratios from the 10 simulated trials were compared against the mean AUC ratio from each in vivo study (fold error). In addition the acceptance criteria proposed by Guest et al. (2011) was also used. This is a more sensitive measure of concordance in reflecting absolute changes in AUC, especially when these are small (Guest et al., 2011). Equation 7 and eq. 8 were used to calculate the geometric mean-fold error (GMFE) and the root-mean square error, which were used to assess the precision of the predictions:

(7)
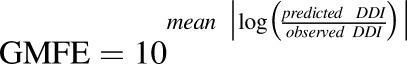


(8)
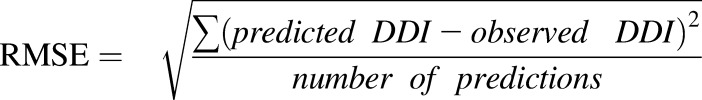


## Results

### 

#### Induction Parameters Determined In Vitro.

**Fig. 2.**
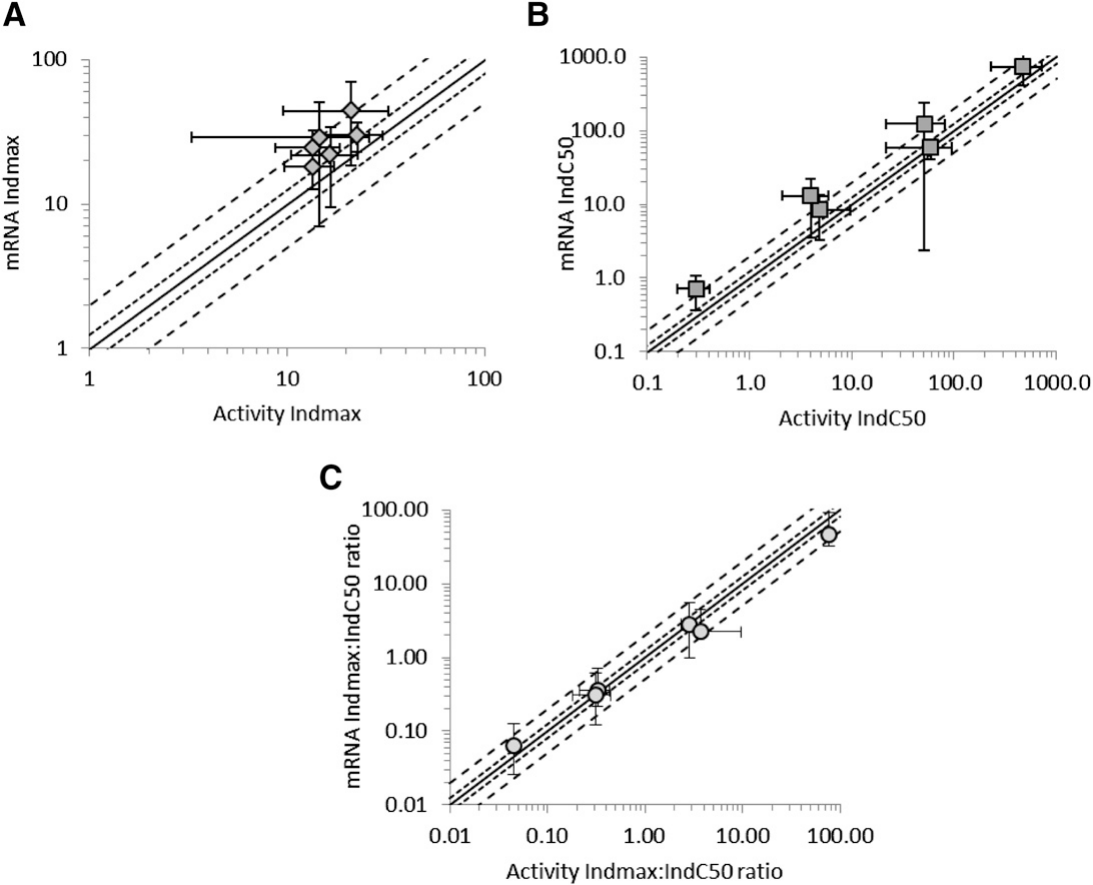
A comparison of *Ind*_max_ (A, diamonds), *IndC*_50_ (B, squares), and the ratio of *Ind*_max_:*IndC*_50_ (C, circles) derived from mRNA and activity data in four human hepatocyte donors (Hu1206, Hu1191, Hu1198, and Hu4193) after incubation with six in vitro inducers of CYP3A (rifampicin, CBZ, PHB, phenytoin, efavirenz, and nifedipine). Data are plotted as mean ± standard deviation. The lines of unity (unbroken line), 0.8- to 1.25-fold (dotted line) and 0.5 to 2-fold (dashed line) are shown.

The in vitro parameters (*Ind*_max_ and *IndC*_50_) for the inducers investigated are shown in Fig. 2 and Table 2. Comparison of the data derived from assessment of mRNA versus activity showed that efficacy was higher (1.3- to 2.0-fold higher *Ind*_max_ values; Fig. 2A), but potency (*IndC*_50_) was generally lower (1.0- to 3.3-fold_;_ Fig. 2B) when measured by changes in mRNA levels compared with changes in activity. When the ratio of *Ind*_max_ to *Ind*C_50_ was compared, no systematic trend was seen for a higher or lower value for mRNA versus activity with fold difference between the two ranging from 0.6- to 1.3-fold (Fig. 2C).

#### Simulations Using the Rifampicin Base Model (Model A; *Ind*_max_ 8, *IndC*_50_ 0.32 *µ*M).

The data in Table 3 show that both the magnitude of interaction and the variability between studies were higher when MDZ was administered orally compared with i.v. administration (median, 17.5-fold; range, 8.0- to 64 vs. 2.0-fold (1.5- to 2.6-fold) reduction in MDZ AUC).

Simulations of the clinical studies describing the changes in exposure of i.v. administered MDZ, before and after multiple dosing with rifampicin, using the default settings in the rifampicin compound file (model A) were in good agreement with the observed data (GMFE 1.21).

Simulated studies describing the effect of multiple dosing of rifampicin on orally administered MDZ exposure predicted a higher fold change in exposure compared with i.v. administered MDZ (median fold change 6.5- versus 1.7-fold), in line with the observed situation (median fold change 18.1- versus 2.0-fold); however, the magnitude of interaction was underpredicted for all clinical studies (GMFE 2.12), despite the wide variability between the clinical studies (range of 1/AUC ratios 8.0–64.3).

**Fig. 3.**
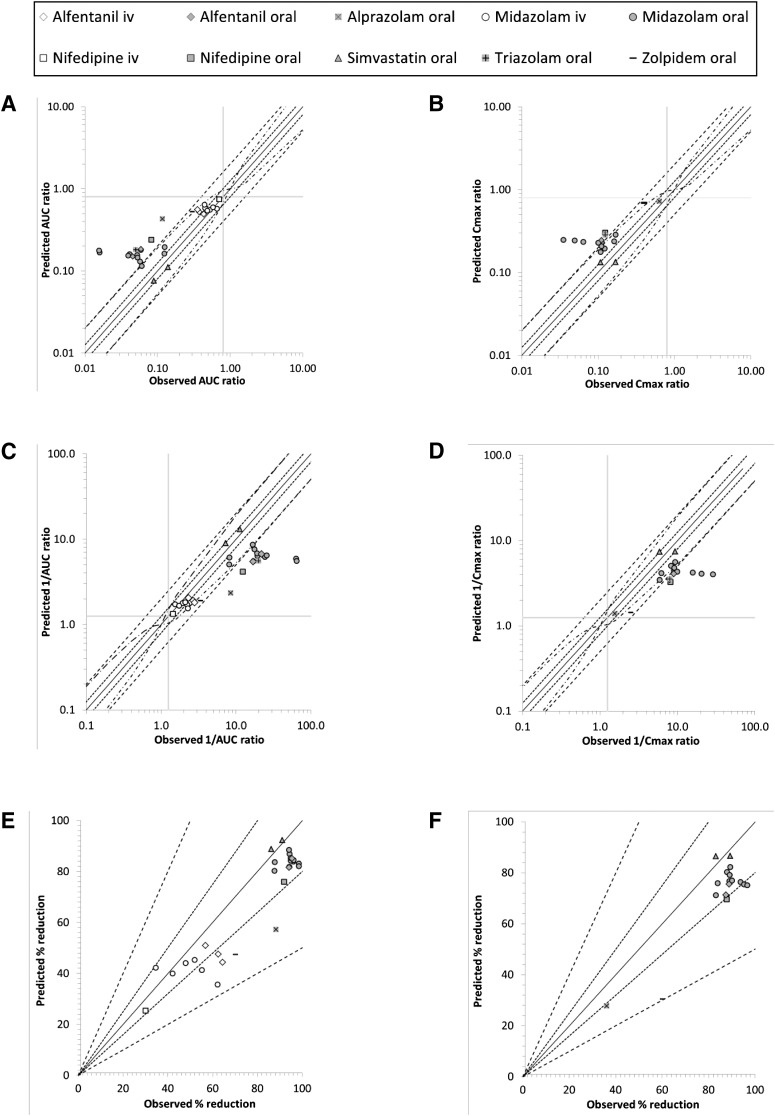
A comparison of the observed and predicted (model A) magnitude of induction for the AUC (A, C, E) and *C*_max_ (B, D, F) of midazolam (circles), nifedipine (squares), alfentanil (diamonds), triazolam (plus sign), alprazolam (cross), zolpidem (dash), and simvastatin (triangles) after their i.v. (open) and oral (closed) administration after multiple doses of rifampicin. Data are plotted as the interaction ratio (A, B), the reciprocal of the interaction ratio (C, D) and as percentage reduction in AUC (E) and *C*_max_ (F). The lines of unity (unbroken line), 0.8- to 1.25-fold (dotted line), 0.5- to 2.0-fold (dashed line), and more cautious limits as suggested by Guest et al. (2011) (broken and dotted line) are shown. Solid vertical and horizontal lines mark 0.8- (A, B) and 1.25- (C, D) fold to show the clinical cutoffs for a DDI.

Plotting the data as a percent change from control indicates excellent prediction accuracy (Fig. 3, E and F), with all predictions for oral MDZ dosing falling between 0.8- and 1.25-fold of the observed value; however, comparison of these data as an interaction ratio or the reciprocal of the ratio show that this is not the case (Fig. 3, A–D).

#### Verification of Simulated Systemic Rifampicin Concentrations and Victim Drug Properties.

Although rifampicin concentrations were not reported for any of the clinical DDI studies (Table 3), independent studies describing the pharmacokinetics of rifampicin in healthy white volunteers were identified and simulated. The predicted plasma concentration-time profiles for rifampicin after multiple dose administration were in reasonable agreement with the observed (Supplemental Fig. 1). Owing to a lack of information describing the metabolism of rifampicin, the model used for rifampicin cannot account for autoinduction, and hence the concentrations of the initial doses were under predicted. This was deemed acceptable as here the focus was on predictions after multiple doses of rifampicin. Simulated key properties (*fm* and *F*_G_) were also in reasonable agreement with those that we observed. (Supplemental Fig. 2)

#### Simulations Using the Modified Rifampicin Models (Models B–E).

**Fig. 4.**
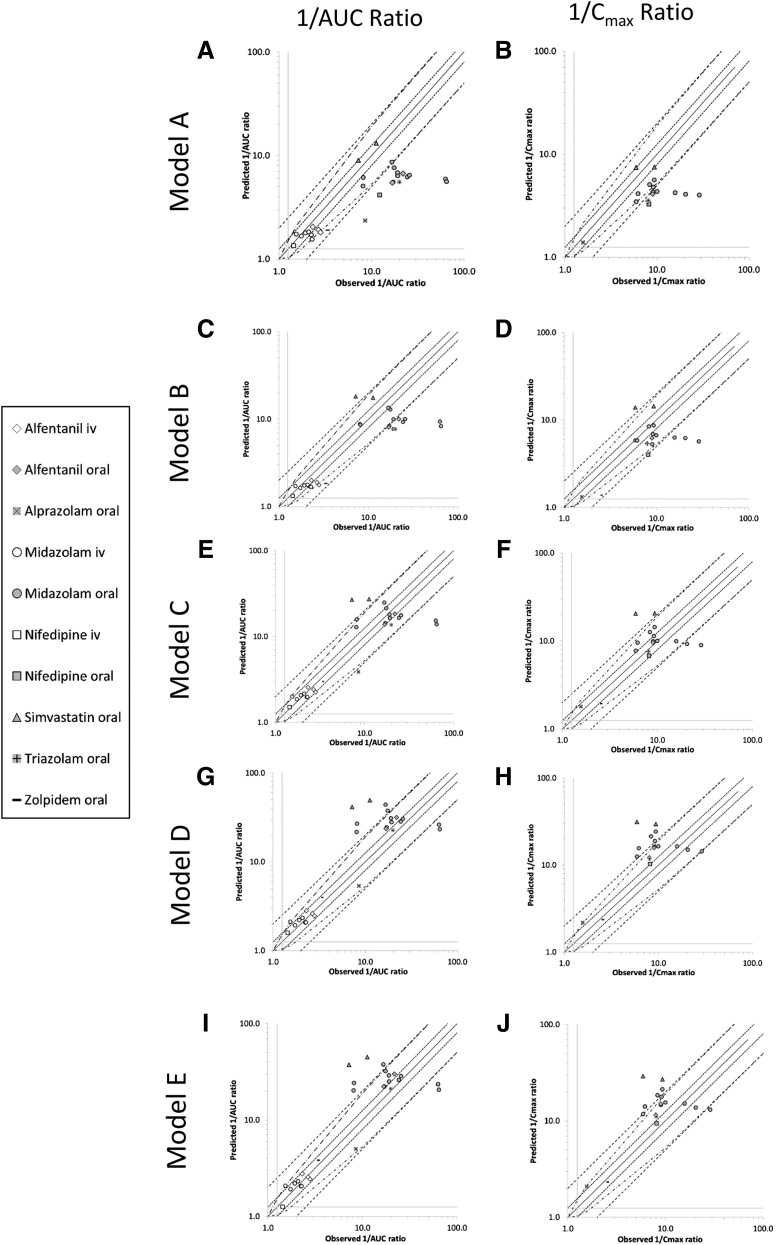
A comparison of the observed and predicted magnitude of induction on the AUC (A, C, E, G, I) and *C*_max_ (B, D, F, H, I) of midazolam (circles), nifedipine (squares), alfentanil (diamonds), triazolam (plus sign), alprazolam (cross) and simvastatin (triangles) after their i.v. (open) and oral (closed) administration after multiple doses of rifampicin (600 mg daily). Predictions were made with models A (A, B), model B (C, D), model C (E, F), model D (G, H), and model E (I, J). Data are plotted as the reciprocal of the interaction ratio. The lines of unity (unbroken line), 0.8- to 1.25-fold (dotted line), 0.5- to 2.0-fold (dashed line), and more cautious limits as suggested by Guest et al. (2011) (broken and dotted line) are shown. Solid vertical and horizontal lines mark 0.8-fold (A, B) and 1.25-fold (C, D) to show the clinical cutoffs for a DDI.

The accuracy of the rifampicin DDI simulations before and after modifications to the base model are described in Table 5 and plotted in Fig. 4. All the alternative models performed better than the base model but to varying degrees. Model B (where *Ind*_max_ for the gut was increased to 16, but Ind_max_ in the liver was kept at 8) improved the predictions (GMFE 1.77 versus 2.12) but not as much as model C (where *Ind*_max_ was changed to 16 in both the liver and the gut; GMFE 1.48 versus 2.12). The highest proportion of predictions to fall within the stringent criteria (Guest et al., 2011) was with models C and F (79.3% of cases). In this study, the uncalibrated assessment of induction using mRNA and activity yielded predictions that were also more accurate than the base model A (1.61 and 1.53 GMFE and 65.5% and 65.5% within acceptance limits for model D activity and model E mRNA, respectively). Additional tested *Ind*_max_ values of 12 (model F) and 20 (model G) also improved the model compared with the base model (1.63 and 1.51 vs. 2.12, respectively).

**TABLE 5:** Summary of the accuracy of DDI predictions using different rifampicin models (A–G)

	Observed	Model A	Model B	Model C	Model D	Model E	Model F	Model G
		*Ind*_max_ 8,*^a^*	*Ind*_max_ 8 liver, 16 gut,	*Ind*_max_ 16	*Ind*_max_ 22.7	Ind_max_ 29.9	Ind_max_ 12	Ind_max_ 20
		*IndC*_50_ 0.32*^a^*	*IndC*_50_ 0.32	*IndC*_50_ 0.32	*IndC*_50_ 0.30	*IndC*_50_ 0.71	*IndC*_50_ 0.32	*IndC*_50_ 0.32
Rifampicin, i.v. MDZ								
Geometric mean fold induction	1.99	1.71	1.72	2.04	2.13	2.11	1.96	2.15
GMFE		1.21	1.21	1.16	1.18	1.18	1.16	1.20
RMSE		0.51	0.47	0.36	0.38	0.38	0.38	0.40
% Within acceptance limits*^b^*		83.3	100	100	83.3	83.3	100	83.3
Rifampicin, oral MDZ								
Geometric mean fold induction	18.1	6.47	9.69	17.1	29.3	26.6	10.9	23.7
GMFE		3.26	2.21	1.70	1.96	1.85	1.99	1.75
RMSE		27.0	24.8	21.6	21.5	20.7	24.1	20.6
% Within acceptance limits*^b^*		27.3	45.5	72.7	36.4	36.4	63.6	63.6
Rifampicin, all MDZ (i.v. and oral)								
Geometric mean fold induction	n/a							
GMFE		2.30	1.79	1.48	1.64	1.58	1.65	1.53
RMSE		21.7	20.0	17.4	17.3	16.7	19.4	16.5
% Within acceptance limits*^b^*		47.1	58.8	82.4	52.9	52.9	76.5	70.6
Rifampicin, all victims (i.v.)								
Geometric mean fold induction	n/a							
GMFE		1.24	1.24	1.15	1.16	1.13	1.16	1.17
RMSE		0.53	0.56	0.35	0.36	0.35	0.42	0.37
% Within acceptance limits*^b^*		90.0	100	100	90.0	90.0	100	90.0
Rifampicin, all victims (oral)								
Geometric mean fold induction	n/a							
GMFE		2.81	2.12	1.69	1.91	1.80	1.96	1.72
RMSE		21.5	19.9	17.7	20.5	19.2	19.1	18.9
% Within acceptance limits*^b^*		26.3	26.3	68.4	52.6	52.6	73.7	68.4
Rifampicin, all victim drugs								
Geometric mean fold induction	n/a							
GMFE		2.12	1.77	1.48	1.61	1.53	1.63	1.51
RMSE		17.4	16.1	14.4	16.5	15.5	15.5	15.3
% Within acceptance limits*^b^*		48.3	51.7	79.3	65.5	65.5	79.3	75.9

GMFE, geometric mean fold error; n/a, not applicable; RMSE, root mean square error.

^*a*^ Default rifampicin induction parameters (V12). Geometric mean fold induction for observed data were calculated in a meta-analysis using published methodology (Einolf, 2007; Cubitt et al., 2011; Ghobadi et al., 2011; Barter et al., 2013; Supplemental Table 3).

^*b*^ Acceptance limits proposed by Guest et al. (2011).

#### Predicted DDIs with Inducers Other than Rifampicin.

**Fig. 5.**
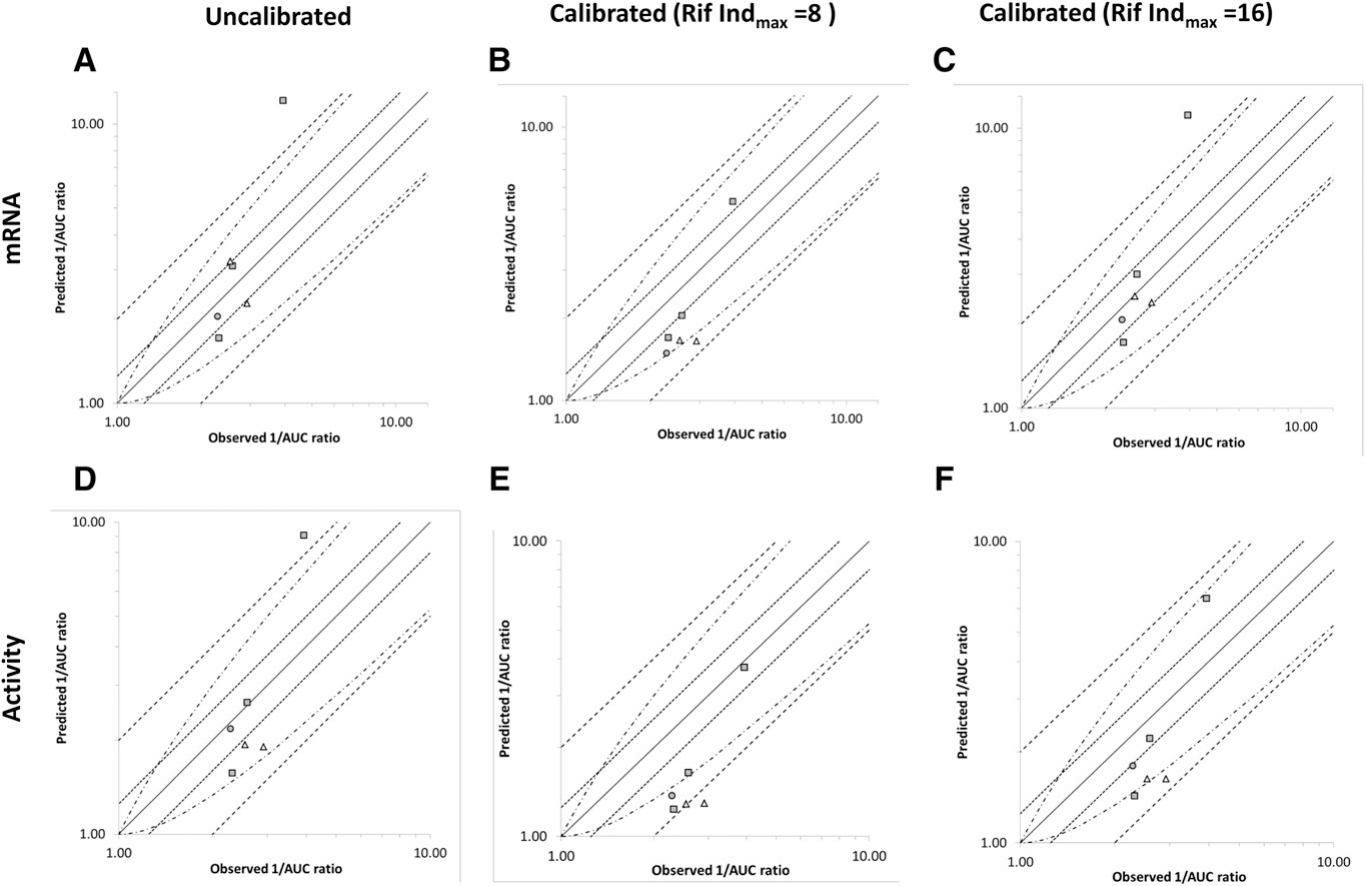
Comparison of the observed and predicted magnitude of change in 1/AUC ratio of orally administered CYP3A4 substrates after administration of multiple doses of CBZ (squares), phenytoin (circles), and PHB (triangles). Predictions are made using in vitro mRNA (A–C) and activity (D–F) data that are uncalibrated (A, D), calibrated using *Ind*_max_ 8, *IndC*_50_ 0.32 (B, E), and calibrated using *Ind*_max_ 16, *IndC*_50_ 0.32. Data are plotted as the reciprocal of the interaction. The lines of unity (unbroken line), 0.8- to 1.25-fold (dotted line), 0.5- to 2.0-fold (dashed line), and more cautious limits as suggested by Guest et al. (2011) (broken and dotted line) are shown. Solid vertical and horizontal lines mark 0.8- (A, B) and 1.25- (C, D) fold to show the clinical cut offs for a DDI.

Simulations for inducers other than rifampicin (CBZ, PHY, and PHB) were run using mRNA and activity data before and after calibration against rifampicin. All calibration was performed using both the original (8) and refined (*16*) Ind_max_ for rifampicin. Comparisons of predicted and observed fold changes in AUC (1/AUC ratio) are shown in Fig. 5. When mRNA data were used to predict the magnitude of induction, the prediction accuracy was similar for uncalibrated, calibrated with an *Ind*_max_ of 8 and calibrated with an *Ind*_max_ of 16, but GMFE was lowest (marginally) when the data were calibrated against an *Ind*_max_ of 16 (Table 6). When activity data were used, calibration against an *Ind*_max_ of 8 gave the lowest prediction accuracy (GMFE 1.7 and 33.3% cases within the acceptance limits). Although predictions with uncalibrated activity data and activity data calibrated against an *Ind*_max_ of 16 were reasonably consistent, uncalibrated activity data gave the higher prediction accuracy (GMFE 1.39 vs. 1.49 and % within acceptance limits 83.3% vs. 66.7%).

**TABLE 6:** Summary of the predication accuracy of drug-drug interactions (1/AUC ratio) for the inducers Six studies (carbamazepine, phenytoin, and phenobarbital) using mRNA and activity data, uncalibrated, calibrated against an *Ind*_max_ = 8 and calibrated against *Ind*_max_=16.

	Activity	mRNA	Activity	mRNA	Activity	mRNA
	Uncalibrated	Uncalibrated	Calibrated (8)	Calibrated (8)	Calibrated (16)	Calibrated (16)
GMFE	1.39	1.44	1.68	1.46	1.49	1.35
RMSE	2.19	3.40	1.09	0.97	1.30	2.98
% Within acceptance limits*^a^*	83.3	83.3	33.3	83.3	66.7	83.3

GMFE, geometric mean fold error; RMSE, root mean square error.

^*a*^ Acceptance limits proposed by Guest et al. (2011).

## Discussion

Changes to regulatory guidance from the FDA have promoted a switch in emphasis from measuring activity to mRNA for assessment of induction in vitro (EMA, 2012; FDA, 2012). Although mRNA has utility as a sensitive marker, especially in cases where a compound is both an inducer and a mechanism-based inhibitor (Fahmi et al., 2009), the magnitude of mRNA changes can be several-fold greater than for activity for CYP3A4 (Luo et al., 2002; Martin et al., 2008; McGinnity et al., 2009). In this investigation, full concentration-induction relationships for mRNA and activity were derived in the same incubation for five clinical inducers (rifampicin, CBZ, PHY, PHB, and efavirenz) and one drug that induces in vitro but not in vivo (nifedipine).

When using in vitro data to quantitatively predict a clinical DDI, one question to consider is what defines a successful prediction. This may be different early in a drug discovery project when a prediction accuracy of 2- to 3-fold may be acceptable for ranking/compound selection, whereas in the later stages of clinical development, where the goals are to define DDI liability and support clinical trial design, a greater degree of accuracy is required, perhaps within 1.25-fold. We have based our assessments of prediction accuracy on calculated values of GMFE and root mean square error for consistency with the literature in this area and have also used more conservative acceptance limits (Guest et al., 2011). Although often overlooked, the variability observed in the clinic between studies with the same compounds can also impact the ability of an IVIVE approach to successfully predict the magnitude of DDI in all individual studies. Because of variability in in vitro induction experiments, the use of in vivo reference values for a calibrator compound have been recommended for the translation of in vitro induction effects to the in vivo situation (Almond et al., 2009). This approach assumes that the efficacy and potency of an inducer relative to the calibrator is the same in vitro as in vivo. Clearly, if a calibration approach is used, the values used for the in vivo calibration will also impact on whether the DDI predictions are successful. In this study, the accuracy of these in vivo reference values was assessed initially by analyzing the accuracy of DDI prediction with rifampicin before assessment of their performance in calibration for other inducers.

The original base model for rifampicin (model A) used *Ind*_max_ values of 8 in the gut and liver and had a higher prediction accuracy for the DDI between oral rifampicin and i.v. MDZ than when MDZ was also dosed orally. This result could be explained by inaccuracy in the extent of change in the first pass extraction in the liver (*E*_H_) and/or gut (*E*_G_) on dosing with rifampicin or may reflect that with a relatively high extraction compound, such as MDZ, there is a limit on the extent of induction that can be observed when the compound is dosed i.v. as hepatic CL becomes limited by hepatic blood flow.

Several factors were considered as explanations for the under prediction of the DDI between rifampicin and orally administered victim drugs. First, the reference values used to predict in vivo effects of rifampicin were derived from two separate studies, one describing the change in metabolic ratio of an endogenous substrate (cortisol) during rifampicin dosing (Tran et al., 1999) and the other the kinetics of rifampicin (Acocella et al., 1971). Because of the variability in rifampicin pharmacokinetics, it is possible that the plasma concentrations in the two studies were different. Second, monitoring the metabolic ratio of an endogenous compound may not provide information on changes in gut metabolism as it is analogous to using a ratio calculated after i.v. administration. The accuracy of DDI prediction was assessed using a range of models where *Ind*_max_ was increased only in the gut or in both gut and liver, respectively. Although all models improved predictions, model C gave the most accurate predictions when MDZ and other victim drugs (with ranging hepatic and gut extraction) were given orally. Recent investigations have also reported a need for higher *Ind*_max_ for rifampicin of 12.5- (Xia et al., 2014), 14.6-, (Baneyx et al., 2014) and 11.5-fold (Wagner et al., 2015). These values are not dissimilar to the value of 16-fold used here and when used in our model gave comparable prediction accuracy. The current study is the only one to have used the refined rifampicin *Ind*_max_ to calibrate in vitro induction data for other inducers and demonstrate application of this strategy for these compounds within a mechanistic dynamic PBPK model. In addition to the in vivo reference *Ind*_max_ and *IndC*_50_ values for rifampicin, other factors that could potentially explain the underprediction of DDI when rifampicin was administered with oral victim drugs were investigated but not shown to have a MDZ significant impact. These included consideration of: 1) induction of UGT1A4-mediated metabolism, 2) a protein-binding displacement interaction leading to a transient increase in the *fu* of the victim drug and increased first-pass clearance, 3) the sensitivity to different values of first-order rate constants (*k*_degH_ and *k*_degG_) that describe endogenous turnover of active enzyme in the liver and gut (Yang et al., 2008), 4) the impact of disparate regional absorption between the victim and perpetrator along the gastrointestinal tract, and 5) sensitivity to different assumptions of the fraction unbound of drug within enterocytes (*fu*_gut_) that is used to calculate both the *F*_G_ (Yang et al., 2007) and the operational concentration of a perpetrator in the gut (Rowland Yeo et al., 2010), in line with recommendations (Zhao et al., 2012). In the latter investigation, changing rifampicin *fu*_gut_ from 0.19 to 1 gave higher simulated unbound portal vein concentrations, but in both cases the free concentrations exceeded the *IndC*_50_ for rifampicin (0.32 *µ*M) across most of the dosing interval; hence, little effect on predictions was observed. In this investigation, absorption of both perpetrator and victim drugs across regions in the gut was assumed to be uniform and not limited by solubility. Further research is required to fully elucidate the cause of under prediction before a mechanistic derivation of in vivo *Ind*_max_ is possible.

Despite the variability in in vitro assays of cytochrome induction, direct entry of mRNA (model D) and activity (model E) data yielded DDI predictions that were in reasonable agreement with the observed (GMFE 1.61 and 1.53 for models D and E, respectively, compared with 2.12 for the best model). The ratio of *Ind*_max_/*Ind*C_50_ for mRNA and the activity in this study were similar, with a tendency for the mRNA data to have both a higher *Ind*_max_ and *IndC*_50_. Although this approach was successful here, a drawback of this approach is that *Ind*_max_ and *IndC*_50_ are influenced by interindividual variability across different donors. In a previous study from this laboratory using different donors, the difference in *Ind*_max_ between the two experimental endpoints was approximately10-fold (Halladay et al., 2012), whereas other investigators have come to similar conclusions (McGinnity et al., 2009). Considerable effort is required to fully characterize each hepatocyte lot by the generation of full *Ind*_max_ and *Ind*C_50_ data for a number of prototypical inducers to ensure that an uncalibrated approach will be successful for a novel compound. Use of empirical scalars (d-factor) has been proposed for mechanistic static models (Fahmi et al., 2008; Fahmi et al., 2009) to account for any systematic deviation between in vitro and in vivo. In some ways, the subsequent scrutiny and correction of in vitro data against a data set (from the same characterized in vitro system) before entry into models is analogous to the d-factor approach but is within a dynamic model.

The advantages of a calibration-based approach are that it controls for the wide variability that is observed in vitro (such as that noted across independent laboratories) (Einolf et al., 2014); it allows the prospective prediction of DDIs, with less emphasis for full characterization of the in vitro system; and provides flexibility in whether data from mRNA or activity are used. In this investigation, we evaluated the existing (*Ind*_max_ 8) and refined (*Ind*_max_ 16) the rifampicin model for the calibration of the prototypical inducers CBZ, PHY, and PHB and showed calibration with the refined model performed reasonably well.

In summary, we have provided a systematic evaluation of the prediction of DDIs mediated by CYP3A4 induction using a mechanistic dynamic model. Use of a range of CYP3A substrates with i.v. and oral administration allowed correction of underprediction, which was then verified with independent predictions for inducers other than rifampicin. Using a comprehensive data set generated using four hepatocyte donors, we were able to compare the predictions made with mRNA and activity data, both calibrated and uncalibrated. Although we believe that calibration with robust in vivo reference values is helpful to combat donor and laboratory variability, uncalibrated data also performed reasonably well with our data set based on prototypical inducers. Use of an uncalibrated approach requires full characterization of the in vitro induction seen within donors and laboratories with prototypical inducers to give an understanding of how that particular system extrapolates to the in vivo situation.

## Supplementary Material

Data Supplement

## Abbreviations

AUC: area under the curve
CBZ: carbamazepine
DDI: drug-drug interaction, *F*_G_, fraction metabolized in the gut
*fu*: fraction unbound
*fu*_,gut_: fraction unbound in the gut
*fm*: fraction metabolized
GMFE: geometric mean fold error
*Ind*_max_: maximal fold induction
IVIVE: in vitro-in vivo extrapolation
*k*_def,_: rate of enzyme degradation)
MDZ: midazolam
PBPK: physiologically based pharmacokinetic
PHB: phenobarbital
PHY: phenytoin
RMSE: root mean square error
