# Supplementary material for: Prediction of Drug-Drug Interactions Arising from CYP3A induction Using a Physiologically Based Dynamic Model
Source: Drug Metab Dispos. 2016 Jun;44(6):821–32. doi: 10.1124/dmd.115.066845 (PMC4885489; doi:10.1124/dmd.115.066845)
Supplement: Data Supplement [file supp_44_6_821__index.html]

Prediction of drug-drug interactions arising from CYP3A induction using a physiologically-based dynamic model — Prediction of Drug-Drug Interactions Arising from CYP3A induction Using a Physiologically Based Dynamic Model — Prediction of CYP3A4 Induction Using PBPK — Data Supplement 

# Prediction of Drug-Drug Interactions Arising from CYP3A induction Using a Physiologically Based Dynamic Model

## Data Supplement

**Files in this Data Supplement:**

- Supplemental Data -

  Supplemental Table 1 - Input parameters of the victim drugs (substrates) used in simulations

  Supplemental Table 2 - Input parameters of the perpetrator drugs (inducers) used in simulations

  Supplemental Table 3 - Meta-analysis of studies where midazolam (victim drug) was administered orally

  Supplemental Figure 1 - Simulated (line) and observed (open circles) mean systemic concentration time profiles of inducers after multiple dosing...

  Supplemental Figure 2 - Simulated (open) and reported (black) fmCYP3A4 (A) and FG (B) values for the victim drugs used in these analyses

  References
