# Supplementary material for: Prediction of Drug-Drug Interactions Arising from CYP3A induction Using a Physiologically Based Dynamic Model
Source: Drug Metab Dispos. 2016 Jun;44(6):821–32. doi: 10.1124/dmd.115.066845 (PMC4885489; doi:10.1124/dmd.115.066845)
Supplement: Data Supplement [file supp_115.066845_Supplemental_66845.pdf]

Prediction of DDIs arising from CYP3A induction using a physiologically-based dynamic model – SUPPLEMENTARY MATERIAL

Lisa M Almond, Sophie Mukadam, Iain Gardner, Krystle Okialda, Susan Wong, Oliver Hatley, Suzanne Tay, Karen Rowland-Yeo, Masoud Jamei, Amin Rostami-Hodjegan and Jane R Kenny

Drug Metabolism and Disposition

Table 1 – Input parameters of the victim drugs (substrates) used in simulations

Parameter	Value	Reference
Alfentanil		
MW	416.52	
fu	0.12	Meta-analysis (Meuldermans et al., 1982); (Bower and Hull, 1982); (Schuttler and Stoeckel, 1982); (Meistelman et al., 1987); (Roure et al., 1987); (Beaumont et al., 2011); (Lemmens et al., 1992)
B:P	0.66	(Meuldermans et al., 1982)<br>(Beaumont et al., 2011)
logP	2.16	(Mather, 1983)
Compound type	Monoprotic Base	
pKa(s)	6.5	(Meuldermans et al., 1982); (Mather, 1983)
fa – predicted	0.99	Predicted from Caco-2 data (Gertz et al., 2010)
ka (h^{-1}) - predicted	4.8	Predicted from Caco-2 data (Gertz et al., 2010)
Qgut (L/h)	2.2	Optimised to recover observed F_G. (Kharasch and Stubbert, 2013); (Kharasch et al., 2011a); (Kharasch et al., 2004a); (Kharasch et al., 2005); (Kharasch et al., 2007); (Kharasch et al., 2009); (Kharasch et al., 2008); (Kharasch et al., 2012); (Kharasch et al., 2011b)
Caco-2 (Papp A-B)	293	(Gertz et al., 2010)
fu_{gut}	1	Assumed
Vss(L/kg) – predicted (Minimal PBPK)	0.397	Rodgers and Rowland method (Kp scalar 0.45 to recover the observed Vss)
Vss (L/kg) - observed	0.371	Meta-analysis (Bower and Hull, 1982); (Bentley et al., 1983); (Bower and Sear, 1989); (Chauvin et al., 1987); (Ibrahim et al., 2003); (Kharasch et al., 2007); (Kharasch et al., 1999); (Kharasch et al., 1997b); (Kharasch et al., 1997a); (Kharasch et al., 2011a); (Kharasch et al., 2004a); (Kharasch et al., 2011b); (Scott and Stanski, 1987); (Meistelman et al., 1987); (Meuldermans et al., 1988)
CLiv (L/h)	19.9	Meta-analysis (Bovill et al., 1982); (Bower and Hull, 1982); (Bower and Sear, 1989); (Camu et al., 1982); (Ibrahim et al., 2003); (Fragen et al., 1983); (Egan et al., 1996); (Helmers et al., 1984); (Mertens et al., 2001); (Scott and Stanski, 1987); (Kharasch and Stubbert, 2013); (Kharasch et al., 2011a); (Kharasch et al., 2004a); (Kharasch et al., 2005); (Kharasch et al., 2007); (Kharasch et al., 2009);

		(Kharasch et al., 1999); (Kharasch et al., 1997a); (Kharasch et al., 1997b); (Kharasch et al., 2008); (Kharasch et al., 2012); (Kharasch et al., 2011b); (McDonnell et al., 1982); (Meistelman et al., 1987); (Meuldermans et al., 1988); (Phimmasone and Kharasch, 2001); (Roure et al., 1987); (Schuttler and Stoeckel, 1982); (McDonnell et al., 2003); (Bentley et al., 1983)
rCYP3A4 CL_{int} (µL/min/pmol)	0.49	Calculated from CL_{iv} using the Retrograde model and fm_{CYP3A4} = 0.93
Add Clint (µL/min/mg mic protein)	4.37	Calculated using the Retrograde model
CL_R (L/h)	0.06	Meta-analysis (Meuldermans et al., 1988); (Schuttler and Stoeckel, 1982)
Alprazolam		
MW	308.8	
fu	0.29	(Scavone et al., 1988); (Ochs et al., 1986); (Moschitto and Greenblatt, 1983); (Greenblatt et al., 1983)
B:P	0.825	(Obach, 1999) & unpublished experimental data obtained by personal communication
Compound type	Monoprotic Base	
pKa(s)	2.4	
fa	1	Assumed
ka (h-1)	3.56	(Smith et al., 1984); (Lin et al., 1988); (Kirkwood et al., 1991); (Amchin et al., 1998); (Greenblatt et al., 1983)
Qgut (L/h)	15.6	Predicted from PSA
fugut	1	Assumed
PSA (A2)	43.07	Calculated with Marvin
HBD	0	Calculated with Marvin
Vss(L/kg) (Minimal PBPK)	0.99	(Lin et al., 1988); (Wong et al., 1998); (Kaplan et al., 1998); (Amchin et al., 1998);<br>(Stoehr et al., 1984)
CL – Enzyme Kinetics<br>4-hydroxylation		
rCYP3A4 Vmax (µL/min/pmol)	17.5	Meta-analysis (corrected for fumic & ISEF)<br>(Hirota et al., 2001); (Galetin et al., 2004)<br>(Williams et al., 2002)
Km (µM)	256.7	
rCYP3A5 V_{max} (µL/min/pmol)	5.99	
Km (µM)	211.4	
oxylation		
rCYP3A4 V_{max} (µL/min/pmol)	0.8	
Km (µM)	118.2	
rCYP3A5 V_{max} (µL/min/pmol)	2.29	
Km (µM)	205.1	

CL_R (L/h)	0.678	(Fraser et al., 1991)
Midazolam		
MW	325.8	
fu	0.032	Meta-analysis (Allonen et al., 1981); (Thummel et al., 1996); (Greenblatt et al., 1984); (Moschitto and Greenblatt, 1983)
B:P	0.603	(Allonen et al., 1981); (Heizmann et al., 1983); (Bjorkman et al., 2001) & unpublished experimental data obtained by personal communication
Compound type	Ampholyte	
pKa(s)	10.95, 6.2	
fa	1	Assumed
ka (h^{-1})	3	(Allonen et al., 1981)
Qgut (L/h)	14.0	Predicted from Caco-2 data (von Richter et al., 2009)
fugut	1	Assumed
Vss(L/kg) (Minimal PBPK)	1	Meta-analysis (Saari et al., 2006); (Ibrahim et al., 2002); (Wandel et al., 2000); (Schwagmeier et al., 1998); (Heizmann et al., 1983); (Allonen et al., 1981)
Enzyme Kinetics<br>1-hydroxylation<br>rCYP3A4 V_{max} (µL/min/pmol)<br>Km (µM)<br>rCYP3A5 V_{max} (µL/min/pmol)<br>Km (µM)<br>4-hydroxylation<br>rCYP3A4 V_{max} (µL/min/pmol)<br>Km (µM)<br>rCYP3A5 V_{max} (µL/min/pmol)<br>Km (µM)<br>rUGT1A4 V_{max} (µL/min/mg)<br>Km (µM)	 <br>5.23<br>2.16<br>19.7<br>4.16<br><br>5.2<br>31.8<br>4.03<br>34.8<br>445<br>40.3	Meta-analysis, corrected for fumic & ISEF (Emoto et al., 2003); (Galetin et al., 2004); (Huang et al., 2004); (Nakajima et al., 2002); (Soars et al., 2006); (Walsky and Obach, 2004); (Weaver et al., 2003); (Williams et al., 2002); (Hyland et al., 2009)
CL_R (L/h)	0.085	Fe 0.0033 from (Thummel et al., 1996)
Nifedipine		
MW	346.3	
fu	0.039	(Ahsan et al., 1993); (Krecic-Shepard et al., 2000)
B:P	0.685	(Holtbecker et al., 1996) & unpublished experimental data obtained by personal communication
logP	2.69	(Masumoto et al., 1995); (Volgyi et al., 2008); (Lombardo et al., 2000)
Compound type	Monoprotic Base	
pKa(s)	2.82	Marvin, ACD Labs

fa	1	Assumed
ka (h^{-1})	3.67	(Ahsan et al., 1993)
Qgut (L/h)	15.8	Predicted from MDCK data (Polli et al., 2001)
fu_{gut}	0.5	Modified to recover observed F_G
Vss(L/kg) (Minimal PBPK)	0.57	(Raemsch and Sommer, 1983); (Holtbecker et al., 1996); (Foster et al., 1983)
CLiv (L/h)	33.6	(Holtbecker et al., 1996); (Raemsch and Sommer, 1983)
Enzyme Kinetics		
rCYP3A4 V_{max} (µL/min/pmol)	33.8	Calculated using the retrograde model and fm3A4 reported in the literature (Ohno et al., 2007; Foti et al., 2010)
Km (µM)	10.95	Meta-analysis from in vitro data (Carr et al., 2006); (Emoto and Iwasaki, 2007); (Williams et al., 2002; Emoto and Iwasaki, 2006)
Add HLM Clint (µL/min/mg)	133.32	Calculated using the retrograde model and fm3A4 reported in the literature
CL_R (L/h)	negligible	(Raemsch and Sommer, 1983)
Quinidine		
MW	324.4	
fu	0.203	(Mihaly et al., 1987); (Kessler and Perez, 1981); (Hughes et al., 1975); (Kates et al., 1978); (Perez-Mateo and Erill, 1977); (Affrime and Reidenberg, 1975); (Nilsen et al., 1978); (Ochs et al., 1978b); (Woo and Greenblatt, 1979); (Ochs et al., 1980); (Ochs et al., 1978a); (Kessler et al., 1978)
B:P	0.88	(Hughes et al., 1975); (Rodgers and Rowland, 2007) & unpublished experimental data obtained by personal communication
logP	2.88	(Hansch et al., 1995)
Compound type	Diprotic Base	
pKa(s)	4.2, 8.8	(Grube et al., 2009)
fa	1	Assumed
ka (h^{-1})	3	Optimised to recover observed tmax (Edwards et al., 1987); (Laganiere et al., 1996)
Qgut (L/h)	11.7	Predicted from Caco-2 data (von Richter et al., 2009)
fu_{gut}	1	Assumed
Vss(L/kg) – Predicted<br>Minimal PBPK – M1	2.03	Corrected Poulin & Theil, corrected by Berezhkovskiy (Poulin and Theil, 2002); (Berezhkovskiy, 2004)
Enzyme Kinetics		
3-hrdoxylation		Meta-analysis, corrected for fumic (Nielsen et al., 1999)
HLM CYP3A4 CL_{int} (µL/min/mg)	20.7	
N-oxidation		
HLM CYP3A4 CL_{int} (µL/min/mg)	2.6	
HLM CYP2C9 CL_{int} (µL/min/mg)	0.40	
HLM CYP2E1 CL_{int} (µL/min/mg)	0.52	

CL_R (L/h)	1.95	(Darbar et al., 1997)
CYP2D6 Ki (µM)	0.017	(Ching et al., 1995); (Broly et al., 1989); (Lalovic et al., 2004)
CYP3A4 Ki (µM)	40	(Nielsen et al., 1999); (Ngui et al., 2000); (Guengerich et al., 1986), fu_{mic} 0.58 (predicted)
3-OH Quinidine		
MW	340.4	
fu	0.281	Simcyp Simulator
B:P	1	Assumed
logP	2	Calculated (ALogPS website & Marvin)
Compound type	Diprotic Base	
pKa(s)	4.2, 2.8	Calculated (Marvin & Moca)
Vss(L/kg) – Predicted<br>Minimal PBPK – M2	1.28	(Rodgers and Rowland, 2007)
CLpo (L/h)	52.6	(Lecocq et al., 1988); (Vozeh et al., 1985)
CL_R (L/h)	16.7	(Lecocq et al., 1988); (Vozeh et al., 1985)
Simvastatin		
MW	418.57	
fu	0.02	(Vickers et al., 1990)
B:P	1	
logP	4.68	(Hansch et al., 1995)
Compound type	Neutral	
pKa(s)	-	
fa	1	Assumed
ka (h^{-1})	1.43	Fitted to recover concentration-time profile
Qgut (L/h)	8.07	Predicted from Caco-2 data ((Gertz et al., 2010))
fu_{gut}	1	Assumed
Vss(L/kg) (Minimal PBPK)	2.26	Fitted to recover concentration time profiles
Enzyme Kinetics		
HLM-CYP3A4 CL_{int} (µL/min/mg)	1873	Optimised
Add HLM-CYP CL_{int} (µL/min/mg)	1873	Optimised
CL_R (L/h)	0	
Zolpidem		
MW	307.39	
fu	0.08	
B:P	0.76	(Obach, 1999)
Compound type	Monoprotic Base	
pKa(s)	6.16	(Durand et al., 1992)

fa	1	Assumed
ka (h^{-1})	2.25	(Olubodun et al., 2003); (Patat et al., 1994)
Qgut (L/h)	16.3	Predicted from MDCK II data (Mahar Doan et al., 2002)
fu_{gut}	1	Assumed
Vss(L/kg) (Minimal PBPK)	0.68	(Patat et al., 1994)
Enzyme Kinetics		
M3		
rCYP1A2 V_{max} (µL/min/pmol)	7.99	
Km (µM)	38	
rCYP2C9 V_{max} (µL/min/pmol)	17.42	
Km (µM)	103	
rCYP2C19 V_{max} (µL/min/pmol)	0.8	
Km (µM)	133	
rCYP2D6 V_{max} (µL/min/pmol)	1.44	
Km (µM)	190	
rCYP3A4 V_{max} (µL/min/pmol)	15.48	
Km (µM)	123	
rCYP2J2 V_{max} (µL/min/pmol)	1.41	
Km (µM)	340	
M4		(Von Moltke et al., 1999), corrected for fumic & ISEF.
rCYP1A2 V_{max} (µL/min/pmol)	0.777	
Km (µM)	40	
rCYP2C9 V_{max} (µL/min/pmol)	0.888	
Km (µM)	81	
rCYP2D6 V_{max} (µL/min/pmol)	4.68	
Km (µM)	214	
rCYP3A4 V_{max} (µL/min/pmol)	1.41	
Km (µM)	340	
rCYP2J2 V_{max} (µL/min/pmol)	6.86	
Km (µM)	399	
M11		
rCYP3A4 V_{max} (µL/min/pmol)	6.89	
Km (µM)	399	
CL_R (L/h)	0.18	fe = 0.01; (Salva and Costa, 1995).

Table 2 – Input parameters of the perpetrator drugs (inducers) used in simulations

Parameter	Value	Reference
Rifampicin		
MW	823	
fu	0.15	(Burman et al., 2001)
B:P	0.9	(Loos et al., 1985)
logP	3.28	AlogPS website
Compound type	Ampholyte	
pKa(s)	1.7, 7.9	
fa	1	Assumed
ka (h^{-1})	0.51	(Drusano et al., 1986a)
Vss(L/kg) (Minimal PBPK)	0.33	(Loos et al., 1985)
CLiv (L/h)	7	Simcyp Library Value
CL_R (L/h)	1.2	Polk 2001
CYP3A4 Ki (μM)	10.5	(Kajosaari et al., 2005)– corrected for fumic
CYP3A4 Ind_{max} (Fold, Emax +1)	8	Base Model, Fitted from in vivo data. (Tran et al., 1999); (Acocella et al., 1971)
CYP3A5 $IndC_{50}$ (μM)	0.32	
Carbamazepine		
MW	236.3	
fu	0.25	Meta-analysis ((Grimsley et al., 1991); (Di Salle et al., 1974); (Rawlins et al., 1975); (Ramsay et al., 1990); (Riva et al., 1984); (MacKichan and Zola, 1984); (Curran et al., 2011)
B:P	1.07	Meta-analysis ((Bonneton et al., 1992); (de Groot et al., 1984)
logP	2.22	(Zhu et al., 2002); (Lombardo et al., 2000); (Henczi et al., 1995); (Wong et al., 2004); Dal Pozzo, A., et al. 1989; (Wan et al., 2009); (Winiwarter et al., 1998)
Compound type	Neutral	
pKa(s)	-	
fa	0.84	(Tchaparian et al., 2008); (Faigle and Feldmann, 1975); (Levy et al., 1975)
ka (h^{-1})	0.5	(Geradin et al., 1976); (Grimsley et al., 1991); (Olling et al., 1999); (Wong et al., 1983) .
Qgut (L/h)	12.6	Predicted from Peff,man (Winiwarter et al., 1998)
Vss(L/kg) (Minimal PBPK)	0.78	(Ramsay et al., 1990)
Enzyme Kinetics		
rCYP3A4 V_{max} (pmol/min/pmol)	0.72	(Cazali et al., 2003); (Huang et al., 2004)
Km (μM)	180	
rCYP3A5 V_{max} (pmol/min/pmol)	1.44	(Huang et al., 2004)
Km (μM)	332	
rCYP2C8 V_{max} (pmol/min/pmol)	0.03	(Cazali et al., 2003)
Km (μM)	741.7	
rUGT2B7 V_{max} (pmol/min/pmol)	4.18	(Staines et al., 2004)

Km (μM)	25.4	
Add rCYP CL_{int} (μL /min/pmol)	0.010	Added to recover degree of auto-induction via enzymes other than CYP3A4
Add HLM CL_{int} (μL/min/mg)	0.255	Other enzymes were combined from (Pearce et al., 2002)
CL_R (L/h)	0.0084	(Kim et al., 2005)
Induction parameters	mRNA/activity data	Generated as part of this study (Table 2)
Carbamazepine-10,11-epoxide		
MW	252.7	
fu	0.48	Meta-analysis (Ramsay et al., 1990); (Riva et al., 1984); (Riad and Sawchuk, 1998); (MacKichan and Zola, 1984)
B:P	1.53	(Bonneton et al., 1992)
logP	1.44	AlogPS website; PubChem website
Compound type	Neutral	
pKa(s)	-	
Vss(L/kg) (Minimal PBPK)	0.78	Assumed equal to parent
CLpo (L/h)	6.07	(Pisani et al., 1988); (Pisani et al., 1992); (Spina et al., 1988); (Tomson et al., 1983)
CL_R (L/h)	0.14	(Kim et al., 2005)
Induction parameters	Equal to parent	Assumed based on data showing equipotency (Oscarson et al., 2006)
Phenytoin		
MW	252.28	
fu	0.10	Meta-analysis (Odar-Cederlof and Borga, 1974); (Tassaneeyakul et al., 1992); (Ducharme et al., 1995); (Kurata and Wilkinson, 1974); (Gugler et al., 1975); (Bochner et al., 1973)
B:P	0.61	(Kurata and Wilkinson, 1974)
logP	2.47	Meta-analysis (Martinavarro-Dominguez et al., 2002); (Pade and Stavchansky, 1998); (Avdeef et al., 2000); (Taillardat-Bertschinger et al., 2002)
Compound type	Monoprotic Acid	
pKa(s)	8.15	Meta-analysis (Pade and Stavchansky, 1998); (Avdeef et al., 2000); (Taillardat-Bertschinger et al., 2002); (Loeuillet-Ritzler and Faller, 2004)
fa	0.9	(Bochner et al., 1973)
ka (h^{-1})	0.53	(Tassaneeyakul et al., 1992)
Qgut (L/h)	13.2	Predicted from Caco-2 data (Irvine et al., 1999)
Vss(L/kg) (Minimal PBPK)	0.57	Meta-analysis (Gugler et al., 1976); (Odar-Cederlof and Borga, 1974); (Glazko et al., 1969); (Lund et al., 1974); (Perucca et al., 1978)
CLiv (L/h)	1.88	Meta-analysis (Gugler et al., 1976); (Glazko et al., 1969); (Lund et al., 1974); (Perucca et al., 1978)
Enzyme Kinetics		
rCYP2C9 V_{max} (pmol/min/pmol)	0.24	Calculated from CLiv using retrograde model
Km (μM)	4.1	(Rowland et al., 2008)

rCYP2C19 V_{max} (pmol/min/pmol)	1.53	Calculated from CLiv using retrograde model
Km (µM)	36.8	(Giancarlo et al., 2001); (Bajpai et al., 1996)
Add HLM CL_{int} (µL/min/mg)	0.97	Calculated from CLiv using retrograde model
CL_R (L/h)	0.015	fe 0.008 (Kozelka and Hine, 1943); (Borga et al., 1979)
CYP2C9 Ind_{max} (Fold, Emax +1)	10.7	(Sahi et al., 2009)
CYP2C9 $IndC_{50}$ (µM)	9.8	
CYP2B6 Ind_{max} (Fold, Emax +1)	1.9	(Hariparsad et al., 2008)
CYP2B6 $IndC_{50}$ (µM)	15.3	
CYP3A4/5 Ind_{max} (Fold, Emax +1)	mRNA/activity data	Generated as part of this study (Table 2)
CYP3A4/5 $IndC_{50}$ (µM)	mRNA/activity data	Generated as part of this study (Table 2)
Phenobarbital		
MW	232	
fu	0.49	(Wallin and Herngren, 1985); (Ehrnebo and Odar-Cederlof, 1975); (Ehrnebo et al., 1971);(Bender et al., 1975); (Shou et al., 2008)
B:P	0.83	(Ehrnebo and Odar-Cederlof, 1975)
logP	1.47	Drug Bank Database, (Xu et al., 2011)
Compound type	Monoprotic Acid	
pKa(s)	7.3	(Xu et al., 2011)
fa	1	(Varma et al., 2012)
ka (h^{-1})	2	Recovers the concentration-time profile of Wilensky et al., 1982
Vss(L/kg) (Minimal PBPK)	0.54	(Wilensky et al., 1982)
CLiv (L/h)	0.3	(Xu et al., 2011)
CL_R (L/h)	0.07	fe 0.24 (Varma et al., 2012)
CYP3A4/5 Ind_{max} (Fold, Emax +1)	mRNA/activity data	Generated as part of this study (Table 2)
CYP3A4/5 $IndC_{50}$ (µM)	mRNA/activity data	Generated as part of this study (Table 2)

Table 3 Meta-analysis of studies where midazolam (victim drug) was administered orally

Study	1/AUC ratio	Standard Deviation	n
(Backman et al., 1996)	24.3	14.2	10
(Backman et al., 1998)	63.0	16.4	9
(Eap et al., 2004)	19.1	24.3*	4
(Gurley et al., 2006)	17.5	20.3*	19
(Gurley et al., 2008)	16.6	20.2*	16
(Reitman et al., 2011)	8.1	10.1*	11
(Kharasch et al., 2004b)	19.0	20.8*	8
(Gorski et al., 2003)	25.6	12.2*	52
**Weighted Geometric Mean**	**18.1**		
**CI of the observations**	**5.4-60.4**		

**Calculated using previously published methodology (Einolf, 2007; Cubitt et al., 2011; Ghobadi et al., 2011; Barter et al., 2013); 3 of the clinical studies used for assessment of DDI prediction (Floyd et al., 2003; Chung et al., 2006; Link et al., 2008) didn't contain enough information to allow calculation of standard deviation and therefore are not included in the weighted geometric mean.*

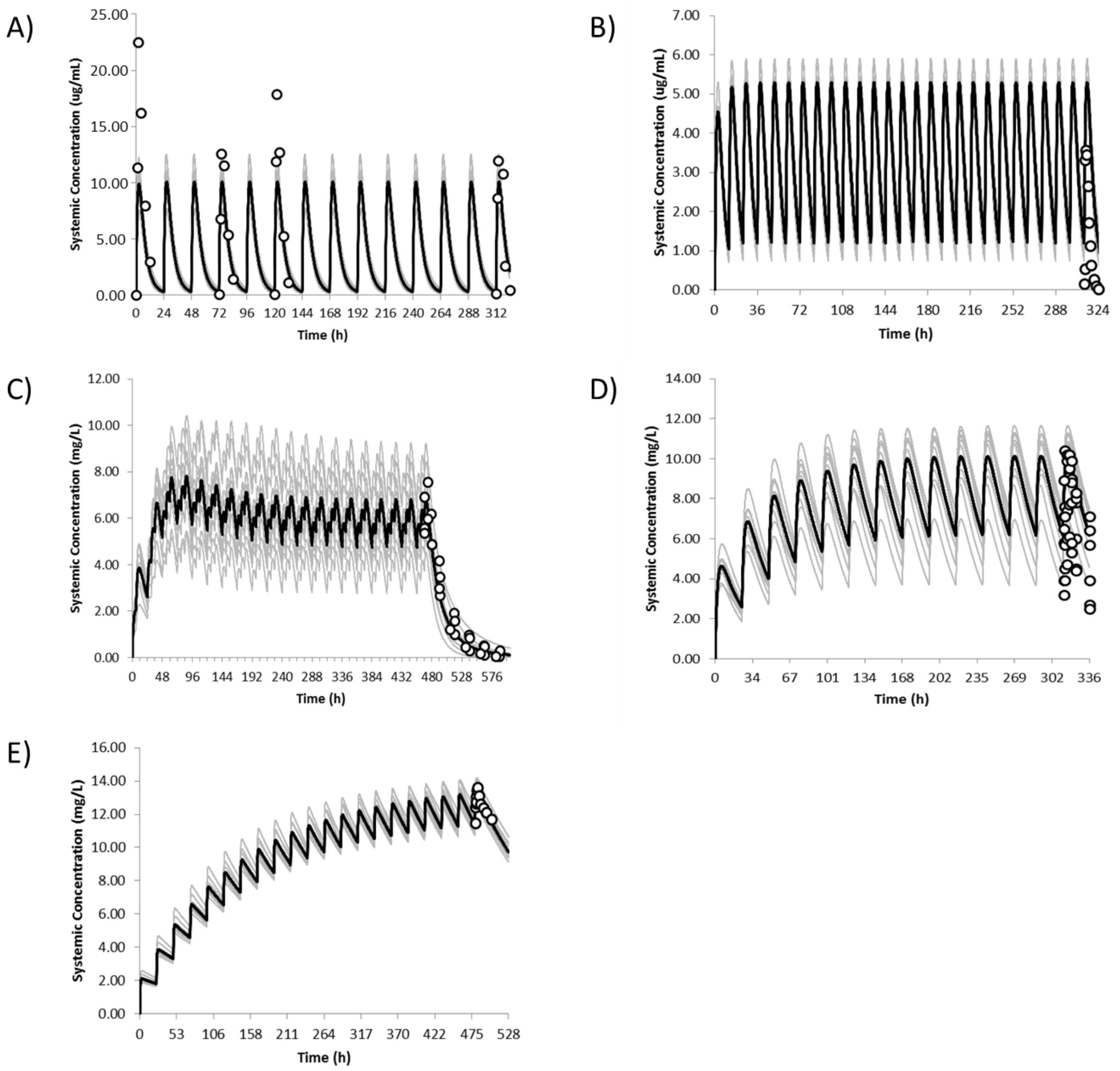

Figure 1 Simulated (line) and observed (open circles) mean systemic concentration time profiles of inducers after multiple dosing of A) rifampicin 600 mg *q.d.* (Acocella et al., 1971), B) rifampicin 300 mg *b.d.* (Drusano et al., 1986b), C) carbamazepine 200 mg *t.i.d* with unequal dosing intervals (Eichelbaum et al., 1975), D) phenytoin 300 mg *q.d.* (Gugler et al., 1976) and E) phenobarbital 90 mg *q.d.* (Ferron et al., 2003).

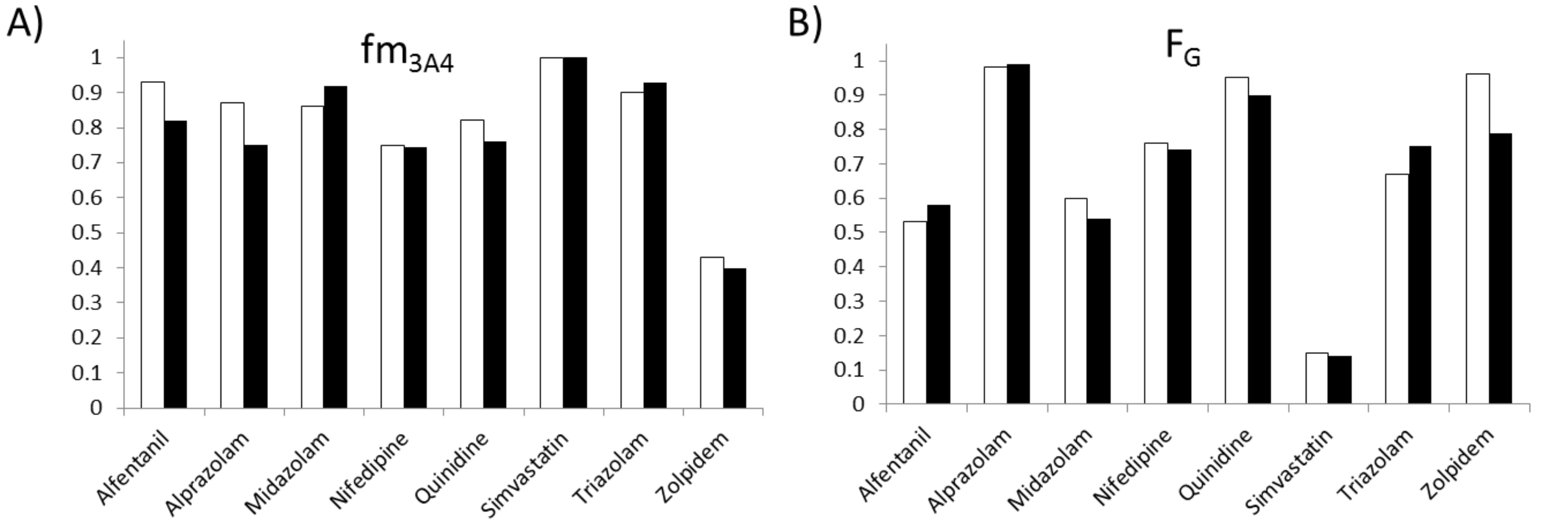

Figure 2 Simulated (open) and reported (black) fm_{CYP3A4} (A) and FG (B) values for the victim drugs used in these analyses. Where multiple reports were available, an average was taken. In the cases of alfentanil and nifedipine a retrograde approach was used to recover the clearance and reported fm and F_G incorporated. Clinical data gave a wide range of fm values (0.72-0.93) for alfentanil but either the inhibitor or trial design was not optimal and hence an fm was derived from in vitro data (0.93, Labroo et al) and verified using a clinical ketoconazole study. For other victim drugs fm_{CYP3A4} is propagated from *in vitro* metabolism data, incorporating other routes of elimination (for fm) and permeability data (for F_G).

REFERENCES

Acocella G, Pagani V, Marchetti M, Baroni GC and Nicolis FB (1971) Kinetic studies on rifampicin. I. Serum concentration analysis in subjects treated with different oral doses over a period of two weeks. *Chemotherapy* **16:**356-370.

Affrime M and Reidenberg MM (1975) The protein binding of some drugs in plasma from patients with alcoholic liver disease. *Eur J Clin Pharmacol* **8:**267-269.

Ahsan CH, Renwick AG, Waller DG, Challenor VF, George CF and Amanullah M (1993) The influence of dose and ethnic origins on the pharmacokinetics of nifedipine. *Clin Pharmacol Ther* **54:**329-338.

Allonen H, Ziegler G and Klotz U (1981) Midazolam kinetics. *Clin Pharmacol Ther* **30:**653-661.

Amchin J, Zarycranski W, Taylor KP, Albano D and Klockowski PM (1998) Effect of venlafaxine on the pharmacokinetics of alprazolam. *Psychopharmacol Bull* **34:**211-219.

Avdeef A, Berger CM and Brownell C (2000) pH-metric solubility. 2: correlation between the acid-base titration and the saturation shake-flask solubility-pH methods. *Pharm Res* **17:**85-89.

Backman JT, Kivisto KT, Olkkola KT and Neuvonen PJ (1998) The area under the plasma concentration-time curve for oral midazolam is 400-fold larger during treatment with itraconazole than with rifampicin. *Eur J Clin Pharmacol* **54:**53-58.

Backman JT, Olkkola KT and Neuvonen PJ (1996) Rifampin drastically reduces plasma concentrations and effects of oral midazolam. *Clin Pharmacol Ther* **59:**7-13.

Bajpai M, Roskos LK, Shen DD and Levy RH (1996) Roles of cytochrome P4502C9 and cytochrome P4502C19 in the stereoselective metabolism of phenytoin to its major metabolite. *Drug Metab Dispos* **24:**1401-1403.

Barter ZE, Tucker GT and Rowland-Yeo K (2013) Differences in cytochrome p450-mediated pharmacokinetics between chinese and caucasian populations predicted by mechanistic physiologically based pharmacokinetic modelling. *Clin Pharmacokinet* **52:**1085-1100.

Beaumont K, Gardner I, Chapman K, Hall M and Rowland M (2011) Toward an integrated human clearance prediction strategy that minimizes animal use. *J Pharm Sci* **100:**4518-4535.

Bender AD, Post A, Meier JP, Higson JE and Reichard G, Jr. (1975) Plasma protein binding of drugs as a function of age in adult human subjects. *J Pharm Sci* **64:**1711-1713.

Bentley JB, Finlay JH, Humphrey LR, Gandolfi AJ and Brown BR (1983) Obesity and Alfentanil Pharmacokinetics *Anesth Analg* **62:**251.

Berezhkovskiy LM (2004) Volume of distribution at steady state for a linear pharmacokinetic system with peripheral elimination. *J Pharm Sci* **93:**1628-1640.

Bjorkman S, Wada DR, Berling BM and Benoni G (2001) Prediction of the disposition of midazolam in surgical patients by a physiologically based pharmacokinetic model. *J Pharm Sci* **90:**1226-1241.

Bochner F, Hooper WD, Sutherland JM, Eadie MJ and Tyrer JH (1973) The renal handling of diphenylhydantoin and 5-(p-hydroxyphenyl)-5-phenylhydantoin. *Clin Pharmacol Ther* **14:**791-796.

Bonneton J, Genton P and Mesdjian E (1992) Distribution of carbamazepine and its epoxide in blood compartments in adolescent and adult epileptic patients. *Biopharm Drug Dispos* **13:**411-416.

Borga O, Hoppel C, Odar-Cederlof I and Garle M (1979) Plasma levels and renal excretion of phenytoin and its metabolites in patients with renal failure. *Clin Pharmacol Ther* **26:**306-314.

Bovill JG, Sebel PS, Blackburn CL and Heykants J (1982) The pharmacokinetics of alfentanil (R39209): a new opioid analgesic. *Anesthesiology* **57:**439-443.

Bower S and Hull CJ (1982) Comparative pharmacokinetics of fentanyl and alfentanil. *Br J Anaesth* **54:**871-877.

Bower S and Sear JW (1989) Disposition of alfentanil in patients receiving a renal transplant. *J Pharm Pharmacol* **41:**654-657.

Broly F, Libersa C, Lhermitte M, Bechtel P and Dupuis B (1989) Effect of quinidine on the dextromethorphan O-demethylase activity of microsomal fractions from human liver. *Br J Clin Pharmacol* **28:**29-36.

Burman WJ, Gallicano K and Peloquin C (2001) Comparative pharmacokinetics and pharmacodynamics of the rifamycin antibacterials. *Clin Pharmacokinet* **40:**327-341.

Camu F, Gepts E, Rucquoi M and Heykants J (1982) Pharmacokinetics of alfentanil in man. *Anesth Analg* **61:**657-661.

Carr B, Norcross R, Fang Y, Lu P, Rodrigues AD, Shou M, Rushmore T and Booth-Genthe C (2006) Characterization of the rhesus monkey CYP3A64 enzyme: species comparisons of CYP3A substrate specificity and kinetics using baculovirus-expressed recombinant enzymes. *Drug Metab Dispos* **34:**1703-1712.

Cazali N, Tran A, Treluyer JM, Rey E, d'Athis P, Vincent J and Pons G (2003) Inhibitory effect of stiripentol on carbamazepine and saquinavir metabolism in human. *Br J Clin Pharmacol* **56:**526-536.

Chauvin M, Lebrault C, Levron JC and Duvaldestin P (1987) Pharmacokinetics of alfentanil in chronic renal failure. *Anesth Analg* **66:**53-56.

Ching MS, Blake CL, Ghabrial H, Ellis SW, Lennard MS, Tucker GT and Smallwood RA (1995) Potent inhibition of yeast-expressed CYP2D6 by dihydroquinidine, quinidine, and its metabolites. *Biochem Pharmacol* **50:**833-837.

Chung E, Nafziger AN, Kazierad DJ and Bertino JS, Jr. (2006) Comparison of midazolam and simvastatin as cytochrome P450 3A probes. *Clin Pharmacol Ther* **79:**350-361.

Cubitt HE, Yeo KR, Howgate EM, Rostami-Hodjegan A and Barter ZE (2011) Sources of interindividual variability in IVIVE of clearance: an investigation into the prediction of benzodiazepine clearance using a mechanistic population-based pharmacokinetic model. *Xenobiotica* **41:**623-638.

Curran RE, Claxton CR, Hutchison L, Harradine PJ, Martin IJ and Littlewood P (2011) Control and measurement of plasma pH in equilibrium dialysis: influence on drug plasma protein binding. *Drug Metab Dispos* **39:**551-557.

Darbar D, Dell'Orto S, Morike K, Wilkinson GR and Roden DM (1997) Dietary salt increases first-pass elimination of oral quinidine. *Clin Pharmacol Ther* **61:**292-300.

de Groot G, van Heijst AN and Maes RA (1984) Charcoal hemoperfusion in the treatment of two cases of acute carbamazepine poisoning. *J Toxicol Clin Toxicol* **22:**349-362.

Di Salle E, Pacifici GM and Morselli PL (1974) Studies on plasma protein binding of carbamazepine. *Pharmacol Res Commun* **6:**193-202.

Drusano GL, Townsend RJ, Walsh TJ, Forrest A, Antal EJ and Standiford HC (1986a) Steady-state serum pharmacokinetics of novobiocin and rifampin alone and in combination. *Antimicrob Agents Chemother* **30:**42-45.

Drusano GL, Townsend RJ, Walsh TJ, Forrest A, Antal EJ and Standiford HC (1986b) Steady-state serum pharmacokinetics of novobiocin and rifampin alone and in combination. *Antimicrob Agents Chemother* **30:**42-45.

Ducharme MP, Slaughter RL, Warbasse LH, Chandrasekar PH, Van de Velde V, Mannens G and Edwards DJ (1995) Itraconazole and hydroxyitraconazole serum concentrations are reduced more than tenfold by phenytoin. *Clin Pharmacol Ther* **58:**617-624.

Durand A, Thenot JP, Bianchetti G and Morselli PL (1992) Comparative pharmacokinetic profile of two imidazopyridine drugs: zolpidem and alpidem. *Drug Metab Rev* **24:**239-266.

Eap CB, Buclin T, Cucchia G, Zullino D, Hustert E, Bleiber G, Golay KP, Aubert AC, Baumann P, Telenti A and Kerb R (2004) Oral administration of a low dose of midazolam (75 microg) as an in vivo probe for CYP3A activity. *Eur J Clin Pharmacol* **60:**237-246.

Edwards DJ, Lavoie R, Beckman H, Blevins R and Rubenfire M (1987) The effect of coadministration of verapamil on the pharmacokinetics and metabolism of quinidine. *Clin Pharmacol Ther* **41:**68-73.

Egan TD, Minto CF, Hermann DJ, Barr J, Muir KT and Shafer SL (1996) Remifentanil versus alfentanil: comparative pharmacokinetics and pharmacodynamics in healthy adult male volunteers. *Anesthesiology* **84:**821-833.

Ehrnebo M, Agurell S, Jalling B and Boreus LO (1971) Age differences in drug binding by plasma proteins: studies on human foetuses, neonates and adults. *Eur J Clin Pharmacol* **3:**189-193.

Ehrnebo M and Odar-Cederlof I (1975) Binding of amobarbital, pentobarbital and diphenylhydantoin to blood cells and plasma proteins in healthy volunteers and uraemic patients. *Eur J Clin Pharmacol* **8:**445-453.

Einolf HJ (2007) Comparison of different approaches to predict metabolic drug-drug interactions. *Xenobiotica* **37:**1257-1294.

Emoto C and Iwasaki K (2006) Enzymatic characteristics of CYP3A5 and CYP3A4: a comparison of in vitro kinetic and drug-drug interaction patterns. *Xenobiotica* **36:**219-233.

Emoto C and Iwasaki K (2007) Approach to predict the contribution of cytochrome P450 enzymes to drug metabolism in the early drug-discovery stage: the effect of the expression of cytochrome b(5) with recombinant P450 enzymes. *Xenobiotica* **37:**986-999.

Emoto C, Murase S, Sawada Y, Jones BC and Iwasaki K (2003) In vitro inhibitory effect of 1-aminobenzotriazole on drug oxidations catalyzed by human cytochrome P450 enzymes: a comparison with SKF-525A and ketoconazole. *Drug Metab Pharmacokinet* **18:**287-295.

Faigle JW and Feldmann KF (1975) Pharmacokinetic Data of Carbamazepine and Its Major Metabolites in Man, in: *Clinical Pharmacology of Anti-Epileptic Drugs* (Schneider H, Janz D, Gardner-Thorpe C, Meinardi H and Sherwin AL eds), pp 159-165, Springer Berlin Heidelberg.

Floyd MD, Gervasini G, Masica AL, Mayo G, George AL, Jr., Bhat K, Kim RB and Wilkinson GR (2003) Genotype-phenotype associations for common CYP3A4 and CYP3A5 variants in the basal and induced metabolism of midazolam in European- and African-American men and women. *Pharmacogenetics* **13:**595-606.

Foster TS, Hamann SR, Richards VR, Bryant PJ, Graves DA and McAllister RG (1983) Nifedipine kinetics and bioavailability after single intravenous and oral doses in normal subjects. *J Clin Pharmacol* **23:**161-170.

Foti RS, Rock DA, Wienkers LC and Wahlstrom JL (2010) Selection of alternative CYP3A4 probe substrates for clinical drug interaction studies using in vitro data and in vivo simulation. *Drug Metab Dispos* **38:**981-987.

Fragen RJ, Booij LH, Braak GJ, Vree TB, Heykants J and Crul JF (1983) Pharmacokinetics of the infusion of alfentanil in man. *Br J Anaesth* **55:**1077-1081.

Fraser AD, Bryan W and Isner AF (1991) Urinary screening for alprazolam and its major metabolites by the Abbott ADx and TDx analyzers with confirmation by GC/MS. *J Anal Toxicol* **15:**25-29.

Galetin A, Brown C, Hallifax D, Ito K and Houston JB (2004) Utility of recombinant enzyme kinetics in prediction of human clearance: impact of variability, CYP3A5, and CYP2C19 on CYP3A4 probe substrates. *Drug Metab Dispos* **32:**1411-1420.

Geradin AP, Abadie FV, Campestrini JA and Theobald W (1976) Pharmacokinetics of carbamazepine in normal humans after single and repeated oral doses. *J Pharmacokinet Biopharm* **4:**521-535.

Gertz M, Harrison A, Houston JB and Galetin A (2010) Prediction of human intestinal first-pass metabolism of 25 CYP3A substrates from in vitro clearance and permeability data. *Drug Metab Dispos* **38:**1147-1158.

Ghobadi C, Johnson TN, Aarabi M, Almond LM, Allabi AC, Rowland-Yeo K, Jamei M and Rostami-Hodjegan A (2011) Application of a systems approach to the bottom-up assessment of pharmacokinetics in obese patients: expected variations in clearance. *Clin Pharmacokinet* **50:**809-822.

Giancarlo GM, Venkatakrishnan K, Granda BW, von Moltke LL and Greenblatt DJ (2001) Relative contributions of CYP2C9 and 2C19 to phenytoin 4-hydroxylation in vitro: inhibition by sulfaphenazole, omeprazole, and ticlopidine. *Eur J Clin Pharmacol* **57:**31-36.

Glazko AJ, Chang T, Baukema J, Dill WA, Goulet JR and Buchanan RA (1969) Metabolic disposition of diphenylhydantoin in normal human subjects following intravenous administration. *Clin Pharmacol Ther* **10:**498-504.

Gorski JC, Vannaprasaht S, Hamman MA, Ambrosius WT, Bruce MA, Haehner-Daniels B and Hall SD (2003) The effect of age, sex, and rifampin administration on intestinal and hepatic cytochrome P450 3A activity. *Clin Pharmacol Ther* **74:**275-287.

Greenblatt DJ, Abernethy DR, Locniskar A, Harmatz JS, Limjuco RA and Shader RI (1984) Effect of age, gender, and obesity on midazolam kinetics. *Anesthesiology* **61:**27-35.

Greenblatt DJ, Divoll M, Abernethy DR, Moschitto LJ, Smith RB and Shader RI (1983) Alprazolam kinetics in the elderly. Relation to antipyrine disposition. *Arch Gen Psychiatry* **40:**287-290.

Grimsley SR, Jann MW, Carter JG, D'Mello AP and D'Souza MJ (1991) Increased carbamazepine plasma concentrations after fluoxetine coadministration. *Clin Pharmacol Ther* **50:**10-15.

Grube S, Langguth P, Junginger HE, Kopp S, Midha KK, Shah VP, Stavchansky S, Dressman JB and Barends DM (2009) Biowaiver monographs for immediate release solid oral dosage forms: quinidine sulfate. *J Pharm Sci* **98:**2238-2251.

Guengerich FP, Muller-Enoch D and Blair IA (1986) Oxidation of quinidine by human liver cytochrome P-450. *Mol Pharmacol* **30:**287-295.

Gugler R, Azarnoff DL and Shoeman DW (1975) Diphenylhydantoin: correlation between protein binding and albumin concentration. *Klin Wochenschr* **53:**445-446.

Gugler R, Manion CV and Azarnoff DL (1976) Phenytoin: pharmacokinetics and bioavailability. *Clin Pharmacol Ther* **19:**135-142.

Gurley B, Hubbard MA, Williams DK, Thaden J, Tong Y, Gentry WB, Breen P, Carrier DJ and Cheboyina S (2006) Assessing the clinical significance of botanical supplementation on human cytochrome P450 3A activity: comparison of a milk thistle and black cohosh product to rifampin and clarithromycin. *J Clin Pharmacol* **46:**201-213.

Gurley BJ, Swain A, Hubbard MA, Hartsfield F, Thaden J, Williams DK, Gentry WB and Tong Y (2008) Supplementation with goldenseal (Hydrastis canadensis), but not kava kava (Piper methysticum), inhibits human CYP3A activity in vivo. *Clin Pharmacol Ther* **83:**61-69.

Hansch C, Leo A and Hoekman D (1995) *Exploring QSAR - Hydrophobic, Electronic, and Steric Constants*. American Chemical Society, Washington, DC.

Hariparsad N, Carr BA, Evers R and Chu X (2008) Comparison of immortalized Fa2N-4 cells and human hepatocytes as in vitro models for cytochrome P450 induction. *Drug Metab Dispos* **36:**1046-1055.

Heizmann P, Eckert M and Ziegler WH (1983) Pharmacokinetics and bioavailability of midazolam in man. *Br J Clin Pharmacol* **16 Suppl 1:**43S-49S.

Helmers H, Van Peer A, Woestenborghs R, Noorduin H and Heykants J (1984) Alfentanil kinetics in the elderly. *Clin Pharmacol Ther* **36:**239-243.

Henczi M, Nagy J and Weaver DF (1995) Determination of octanol-water partition coefficients by an HPLC method for anticonvulsant structure-activity studies. *J Pharm Pharmacol* **47:**345-347.

Hirota N, Ito K, Iwatsubo T, Green CE, Tyson CA, Shimada N, Suzuki H and Sugiyama Y (2001) In vitro/in vivo scaling of alprazolam metabolism by CYP3A4 and CYP3A5 in humans. *Biopharm Drug Dispos* **22:**53-71.

Holtbecker N, Fromm MF, Kroemer HK, Ohnhaus EE and Heidemann H (1996) The nifedipine-rifampin interaction. Evidence for induction of gut wall metabolism. *Drug Metab Dispos* **24:**1121-1123.

Huang W, Lin YS, McConn DJ, 2nd, Calamia JC, Totah RA, Isoherranen N, Glodowski M and Thummel KE (2004) Evidence of significant contribution from CYP3A5 to hepatic drug metabolism. *Drug Metab Dispos* **32:**1434-1445.

Hughes IE, Ilett KF and Jellett LB (1975) The distribution of quinidine in human blood. *Br J Clin Pharmacol* **2:**521-525.

Hyland R, Osborne T, Payne A, Kempshall S, Logan YR, Ezzeddine K and Jones B (2009) In vitro and in vivo glucuronidation of midazolam in humans. *Br J Clin Pharmacol* **67:**445-454.

Ibrahim A, Karim A, Feldman J and Kharasch E (2002) The influence of parecoxib, a parenteral cyclooxygenase-2 specific inhibitor, on the pharmacokinetics and clinical effects of midazolam. *Anesth Analg* **95:**667-673.

Ibrahim AE, Feldman J, Karim A and Kharasch ED (2003) Simultaneous assessment of drug interactions with low- and high-extraction opioids: application to parecoxib effects on the pharmacokinetics and pharmacodynamics of fentanyl and alfentanil. *Anesthesiology* **98:**853-861.

Irvine JD, Takahashi L, Lockhart K, Cheong J, Tolan JW, Selick HE and Grove JR (1999) MDCK (Madin-Darby canine kidney) cells: A tool for membrane permeability screening. *J Pharm Sci* **88:**28-33.

Kajosaari LI, Laitila J, Neuvonen PJ and Backman JT (2005) Metabolism of repaglinide by CYP2C8 and CYP3A4 in vitro: effect of fibrates and rifampicin. *Basic Clin Pharmacol Toxicol* **97:**249-256.

Kaplan GB, Greenblatt DJ, Ehrenberg BL, Goddard JE, Harmatz JS and Shader RI (1998) Single-dose pharmacokinetics and pharmacodynamics of alprazolam in elderly and young subjects. *J Clin Pharmacol* **38:**14-21.

Kates RE, Sokoloski TD and Comstock TJ (1978) Binding of quinidine to plasma proteins in normal subjects and in patients with hyperlipoproteinemias. *Clin Pharmacol Ther* **23:**30-35.

Kessler KM, Humphries WC, Jr., Black M and Spann JF (1978) Quinidine pharmacokinetics in patients with cirrhosis or receiving propranolol. *Am Heart J* **96:**627-635.

Kessler KM and Perez GO (1981) Decreased quinidine plasma protein binding during hemodialysis. *Clin Pharmacol Ther* **30:**121-126.

Kharasch ED, Bedynek PS, Hoffer C, Walker A and Whittington D (2012) Lack of indinavir effects on methadone disposition despite inhibition of hepatic and intestinal cytochrome P4503A (CYP3A). *Anesthesiology* **116:**432-447.

Kharasch ED, Bedynek PS, Walker A, Whittington D and Hoffer C (2008) Mechanism of ritonavir changes in methadone pharmacokinetics and pharmacodynamics: II. Ritonavir effects on CYP3A and P-glycoprotein activities. *Clin Pharmacol Ther* **84:**506-512.

Kharasch ED, Francis A, London A, Frey K, Kim T and Blood J (2011a) Sensitivity of intravenous and oral alfentanil and pupillary miosis as minimal and noninvasive probes for hepatic and first-pass CYP3A induction. *Clin Pharmacol Ther* **90:**100-108.

Kharasch ED, Hoffer C, Whittington D, Walker A and Bedynek PS (2009) Methadone pharmacokinetics are independent of cytochrome P4503A (CYP3A) activity and gastrointestinal drug transport: insights from methadone interactions with ritonavir/indinavir. *Anesthesiology* **110:**660-672.

Kharasch ED, Jubert C, Senn T, Bowdle TA and Thummel KE (1999) Intraindividual variability in male hepatic CYP3A4 activity assessed by alfentanil and midazolam clearance. *J Clin Pharmacol* **39:**664-669.

Kharasch ED, Russell M, Garton K, Lentz G, Bowdle TA and Cox K (1997a) Assessment of cytochrome P450 3A4 activity during the menstrual cycle using alfentanil as a noninvasive probe. *Anesthesiology* **87:**26-35.

Kharasch ED, Russell M, Mautz D, Thummel KE, Kunze KL, Bowdle A and Cox K (1997b) The role of cytochrome P450 3A4 in alfentanil clearance. Implications for interindividual variability in disposition and perioperative drug interactions. *Anesthesiology* **87:**36-50.

Kharasch ED and Stubbert K (2013) Cytochrome P4503A does not mediate the interaction between methadone and ritonavir-lopinavir. *Drug Metab Dispos* **41:**2166-2174.

Kharasch ED, Vangveravong S, Buck N, London A, Kim T, Blood J and Mach RH (2011b) Concurrent assessment of hepatic and intestinal cytochrome P450 3A activities using deuterated alfentanil. *Clin Pharmacol Ther* **89:**562-570.

Kharasch ED, Walker A, Hoffer C and Sheffels P (2004a) Intravenous and oral alfentanil as in vivo probes for hepatic and first-pass cytochrome P450 3A activity: noninvasive assessment by use of pupillary miosis. *Clin Pharmacol Ther* **76:**452-466.

Kharasch ED, Walker A, Hoffer C and Sheffels P (2004b) Intravenous and oral alfentanil as in vivo probes for hepatic and first-pass cytochrome P450 3A activity: noninvasive assessment by use of pupillary miosis. *Clin Pharmacol Ther* **76:**452-466.

Kharasch ED, Walker A, Hoffer C and Sheffels P (2005) Sensitivity of intravenous and oral alfentanil and pupillary miosis as minimally invasive and noninvasive probes for hepatic and first-pass CYP3A activity. *J Clin Pharmacol* **45:**1187-1197.

Kharasch ED, Walker A, Isoherranen N, Hoffer C, Sheffels P, Thummel K, Whittington D and Ensign D (2007) Influence of CYP3A5 genotype on the pharmacokinetics and pharmacodynamics of the cytochrome P4503A probes alfentanil and midazolam. *Clin Pharmacol Ther* **82:**410-426.

Kim KA, Oh SO, Park PW and Park JY (2005) Effect of probenecid on the pharmacokinetics of carbamazepine in healthy subjects. *Eur J Clin Pharmacol* **61:**275-280.

Kirkwood C, Moore A, Hayes P, DeVane CL and Pelonero A (1991) Influence of menstrual cycle and gender on alprazolam pharmacokinetics. *Clin Pharmacol Ther* **50:**404-409.

Kozelka FL and Hine CH (1943) Degradation products of Dilantin. *J Pharmacol Exp Ther* **77:**175-179.

Krecic-Shepard ME, Park K, Barnas C, Slimko J, Kerwin DR and Schwartz JB (2000) Race and sex influence clearance of nifedipine: results of a population study. *Clin Pharmacol Ther* **68:**130-142.

Kurata D and Wilkinson GR (1974) Erythrocyte uptake and plasma binding of diphenylhydantoin. *Clin Pharmacol Ther* **16:**355-362.

Laganiere S, Davies RF, Carignan G, Foris K, Goernert L, Carrier K, Pereira C and McGilveray I (1996) Pharmacokinetic and pharmacodynamic interactions between diltiazem and quinidine. *Clin Pharmacol Ther* **60:**255-264.

Lalovic B, Phillips B, Risler LL, Howald W and Shen DD (2004) Quantitative contribution of CYP2D6 and CYP3A to oxycodone metabolism in human liver and intestinal microsomes. *Drug Metab Dispos* **32:**447-454.

Lecocq B, Jaillon P, Lecocq V, Ferry A, Gardin ME, Jarreau C, Leroyer R, Pays M and Jarreau FX (1988) Clinical pharmacology of hydroxy-3(S)-dihydroquinidine in healthy volunteers following oral administration. *J Cardiovasc Pharmacol* **12:**445-450.

Levy RH, Pitlick WH, Troupin AS, Green JR and Neal JM (1975) Pharmacokinetics of carbamazepine in normal man. *Clin Pharmacol Ther* **17:**657-668.

Lin KM, Lau JK, Smith R, Phillips P, Antal E and Poland RE (1988) Comparison of alprazolam plasma levels in normal Asian and Caucasian male volunteers. *Psychopharmacology (Berl)* **96:**365-369.

Link B, Haschke M, Grignaschi N, Bodmer M, Aschmann YZ, Wenk M and Krahenbuhl S (2008) Pharmacokinetics of intravenous and oral midazolam in plasma and saliva in humans: usefulness of saliva as matrix for CYP3A phenotyping. *Br J Clin Pharmacol* **66:**473-484.

Loeuillet-Ritzler F and Faller B (2004) High-Throughput pKa with the Sirius Profiler SGA using a co-solvent approach, in: *3rd Lipophilicity Symposium LogP2004*, Zürich, Switzerland.

Lombardo F, Shalaeva MY, Tupper KA, Gao F and Abraham MH (2000) ElogPoct: a tool for lipophilicity determination in drug discovery. *J Med Chem* **43:**2922-2928.

Loos U, Musch E, Jensen JC, Mikus G, Schwabe HK and Eichelbaum M (1985) Pharmacokinetics of oral and intravenous rifampicin during chronic administration. *Klin Wochenschr* **63:**1205-1211.

Lund L, Alvan G, Berlin A and Alexanderson B (1974) Pharmacokinetics of single and multiple doses of phenytoin in man. *Eur J Clin Pharmacol* **7:**81-86.

MacKichan JJ and Zola EM (1984) Determinants of carbamazepine and carbamazepine 10,11-epoxide binding to serum protein, albumin and alpha 1-acid glycoprotein. *Br J Clin Pharmacol* **18:**487-493.

Mahar Doan KM, Humphreys JE, Webster LO, Wring SA, Shampine LJ, Serabjit-Singh CJ, Adkison KK and Polli JW (2002) Passive permeability and P-glycoprotein-mediated efflux differentiate central nervous system (CNS) and non-CNS marketed drugs. *J Pharmacol Exp Ther* **303:**1029-1037.

Martinavarro-Dominguez A, Capella-Peiro ME, Gil-Agusti M, Marcos-Tomas JV and Esteve-Romero J (2002) Therapeutic drug monitoring of anticonvulsant drugs by micellar HPLC with direct injection of serum samples. *Clin Chem* **48:**1696-1702.

Masumoto K, Takeyasu A, Oizumi K and Kobayashi T (1995) [Studies of novel 1,4-dihydropyridine Ca antagonist CS-905. I. Measurement of partition coefficient (log P) by high performance liquid chromatography (HPLC)]. *Yakugaku Zasshi* **115:**213-220.

Mather LE (1983) Clinical pharmacokinetics of fentanyl and its newer derivatives. *Clin Pharmacokinet* **8:**422-446.

McDonnell CG, Malkan D, Van Pelt FD and Shorten GD (2003) Elimination of alfentanil delivered by infusion is not altered by the chronic administration of atorvastatin. *Eur J Anaesthesiol* **20:**662-667.

McDonnell TE, Bartkowski RR, Bonilla BS, Henthorn TK and Williams JJ (1982) Nonuniformity of Alfentanil pharmacokinetics in healthy adults. *Anesthesiology* **57:**A236.

Meistelman C, Saint-Maurice C, Lepaul M, Levron JC, Loose JP and Mac Gee K (1987) A comparison of alfentanil pharmacokinetics in children and adults. *Anesthesiology* **66:**13-16.

Mertens MJ, Vuyk J, Olofsen E, Bovill JG and Burm AG (2001) Propofol alters the pharmacokinetics of alfentanil in healthy male volunteers. *Anesthesiology* **94:**949-957.

Meuldermans W, Van Peer A, Hendrickx J, Woestenborghs R, Lauwers W, Heykants J, Vanden Bussche G, Van Craeyvelt H and Van der Aa P (1988) Alfentanil pharmacokinetics and metabolism in humans. *Anesthesiology* **69:**527-534.

Meuldermans WE, Hurkmans RM and Heykants JJ (1982) Plasma protein binding and distribution of fentanyl, sufentanil, alfentanil and lofentanil in blood. *Arch Int Pharmacodyn Ther* **257:**4-19.

Mihaly GW, Ching MS, Klejn MB, Paull J and Smallwood RA (1987) Differences in the binding of quinine and quinidine to plasma proteins. *Br J Clin Pharmacol* **24:**769-774.

Moschitto LJ and Greenblatt DJ (1983) Concentration-independent plasma protein binding of benzodiazepines. *J Pharm Pharmacol* **35:**179-180.

Nakajima M, Tane K, Nakamura S, Shimada N, Yamazaki H and Yokoi T (2002) Evaluation of approach to predict the contribution of multiple cytochrome P450s in drug metabolism using relative activity factor: effects of the differences in expression levels of NADPH-cytochrome P450 reductase and cytochrome b(5) in the expression system and the differences in the marker activities. *J Pharm Sci* **91:**952-963.

Ngui JS, Tang W, Stearns RA, Shou M, Miller RR, Zhang Y, Lin JH and Baillie TA (2000) Cytochrome P450 3A4-mediated interaction of diclofenac and quinidine. *Drug Metab Dispos* **28:**1043-1050.

Nielsen TL, Rasmussen BB, Flinois JP, Beaune P and Brosen K (1999) In vitro metabolism of quinidine: the (3S)-3-hydroxylation of quinidine is a specific marker reaction for cytochrome P-4503A4 activity in human liver microsomes. *J Pharmacol Exp Ther* **289:**31-37.

Nilsen OG, Leren P, Aakesson I and Jacobsen S (1978) Binding of quinidine in sera with different levels of triglycerides, cholesterol, and orosomucoid protein. *Biochem Pharmacol* **27:**871-876.

Obach RS (1999) Prediction of human clearance of twenty-nine drugs from hepatic microsomal intrinsic clearance data: An examination of in vitro half-life approach and nonspecific binding to microsomes. *Drug Metab Dispos* **27:**1350-1359.

Ochs HR, Greenblatt DJ, Labedzki L and Smith RB (1986) Alprazolam kinetics in patients with renal insufficiency. *J Clin Psychopharmacol* **6:**292-294.

Ochs HR, Greenblatt DJ, Woo E, Franke K and Smith TW (1978a) Effect of propranolol on pharmacokinetics and acute electrocardiographic changes following intravenous quinidine in humans. *Pharmacology* **17:**301-306.

Ochs HR, Greenblatt DJ, Woo E and Smith TW (1978b) Reduced quinidine clearance in elderly persons. *Am J Cardiol* **42:**481-485.

Ochs HR, Grube E, Greenblatt DJ, Woo E and Bodem G (1980) Intravenous quinidine: pharmacokinetic properties and effects on left ventricular performance in humans. *Am Heart J* **99:**468-475.

Odar-Cederlof I and Borga O (1974) Kinetics of diphenylhydantoin in uraemic patients: consequences of decreased plasma protein binding. *Eur J Clin Pharmacol* **7:**31-37.

Ohno Y, Hisaka A and Suzuki H (2007) General framework for the quantitative prediction of CYP3A4-mediated oral drug interactions based on the AUC increase by coadministration of standard drugs. *Clin Pharmacokinet* **46:**681-696.

Olling M, Mensinga TT, Barends DM, Groen C, Lake OA and Meulenbelt J (1999) Bioavailability of carbamazepine from four different products and the occurrence of side effects. *Biopharm Drug Dispos* **20:**19-28.

Olubodun JO, Ochs HR, von Moltke LL, Roubenoff R, Hesse LM, Harmatz JS, Shader RI and Greenblatt DJ (2003) Pharmacokinetic properties of zolpidem in elderly and young adults: possible modulation by testosterone in men. *Br J Clin Pharmacol* **56:**297-304.

Oscarson M, Zanger UM, Rifki OF, Klein K, Eichelbaum M and Meyer UA (2006) Transcriptional profiling of genes induced in the livers of patients treated with carbamazepine. *Clin Pharmacol Ther* **80:**440-456.

Pade V and Stavchansky S (1998) Link between drug absorption solubility and permeability measurements in Caco-2 cells. *J Pharm Sci* **87:**1604-1607.

Patat A, Trocherie S, Thebault JJ, Rosenzweig P, Dubruc C, Bianchetti G, Court LA and Morselli PL (1994) EEG profile of intravenous zolpidem in healthy volunteers. *Psychopharmacology (Berl)* **114:**138-146.

Pearce RE, Vakkalagadda GR and Leeder JS (2002) Pathways of carbamazepine bioactivation in vitro I. Characterization of human cytochromes P450 responsible for the formation of 2- and 3-hydroxylated metabolites. *Drug Metab Dispos* **30:**1170-1179.

Perez-Mateo M and Erill S (1977) Protein binding of salicylate and quinidine in plasma from patients with renal failure, chronic liver disease and chronic respiratory insufficiency. *Eur J Clin Pharmacol* **11:**225-231.

Perucca E, Makki K and Richens A (1978) Is phenytoin metabolism dose-dependent by enzyme saturation or by feedback inhibition? *Clin Pharmacol Ther* **24:**46-51.

Phimmasone S and Kharasch ED (2001) A pilot evaluation of alfentanil-induced miosis as a noninvasive probe for hepatic cytochrome P450 3A4 (CYP3A4) activity in humans. *Clin Pharmacol Ther* **70:**505-517.

Pisani F, Fazio A, Artesi C, Oteri G, Spina E, Tomson T and Perucca E (1992) Impairment of carbamazepine-10, 11-epoxide elimination by valnoctamide, a valpromide isomer, in healthy subjects. *Br J Clin Pharmacol* **34:**85-87.

Pisani F, Fazio A, Oteri G, Spina E, Perucca E and Bertilsson L (1988) Effect of valpromide on the pharmacokinetics of carbamazepine-10, 11-epoxide. *Br J Clin Pharmacol* **25:**611-613.

Polli JW, Wring SA, Humphreys JE, Huang L, Morgan JB, Webster LO and Serabjit-Singh CS (2001) Rational use of in vitro P-glycoprotein assays in drug discovery. *J Pharmacol Exp Ther* **299:**620-628.

Poulin P and Theil FP (2002) Prediction of pharmacokinetics prior to in vivo studies. 1. Mechanism-based prediction of volume of distribution. *J Pharm Sci* **91:**129-156.

Raemsch KD and Sommer J (1983) Pharmacokinetics and metabolism of nifedipine. *Hypertension* **5:**II18-24.

Ramsay RE, McManus DQ, Guterman A, Briggle TV, Vazquez D, Perchalski R, Yost RA and Wong P (1990) Carbamazepine metabolism in humans: effect of concurrent anticonvulsant therapy. *Ther Drug Monit* **12:**235-241.

Rawlins MD, Collste P, Bertilsson L and Palmer L (1975) Distribution and elimination kinetics of carbamazepine in man. *Eur J Clin Pharmacol* **8:**91-96.

Reitman ML, Chu X, Cai X, Yabut J, Venkatasubramanian R, Zajic S, Stone JA, Ding Y, Witter R, Gibson C, Roupe K, Evers R, Wagner JA and Stoch A (2011) Rifampin's acute inhibitory and chronic inductive drug interactions: experimental and model-based approaches to drug-drug interaction trial design. *Clin Pharmacol Ther* **89:**234-242.

Riad LE and Sawchuk RJ (1998) A partial area difference analysis for estimating elimination rate constants and distribution volumes of metabolites. *J Pharm Sci* **87:**769-773.

Riva R, Contin M, Albani F, Perucca E, Ambrosetto G, Gobbi G, Cortelli P, Procaccianti G and Baruzzi A (1984) Free and total plasma concentrations of carbamazepine and carbamazepine-10,11-epoxide in epileptic patients: diurnal fluctuations and relationship with side effects. *Ther Drug Monit* **6:**408-413.

Rodgers T and Rowland M (2007) Mechanistic approaches to volume of distribution predictions: understanding the processes. *Pharm Res* **24:**918-933.

Roure P, Jean N, Leclerc AC, Cabanel N, Levron JC and Duvaldestin P (1987) Pharmacokinetics of alfentanil in children undergoing surgery. *Br J Anaesth* **59:**1437-1440.

Rowland A, Elliot DJ, Knights KM, Mackenzie PI and Miners JO (2008) The "albumin effect" and in vitro-in vivo extrapolation: sequestration of long-chain unsaturated fatty acids enhances phenytoin hydroxylation by human liver microsomal and recombinant cytochrome P450 2C9. *Drug Metab Dispos* **36:**870-877.

Saari TI, Laine K, Leino K, Valtonen M, Neuvonen PJ and Olkkola KT (2006) Effect of voriconazole on the pharmacokinetics and pharmacodynamics of intravenous and oral midazolam. *Clin Pharmacol Ther* **79:**362-370.

Sahi J, Shord SS, Lindley C, Ferguson S and LeCluyse EL (2009) Regulation of cytochrome P450 2C9 expression in primary cultures of human hepatocytes. *J Biochem Mol Toxicol* **23:**43-58.

Salva P and Costa J (1995) Clinical pharmacokinetics and pharmacodynamics of zolpidem. Therapeutic implications. *Clin Pharmacokinet* **29:**142-153.

Scavone JM, Greenblatt DJ, Locniskar A and Shader RI (1988) Alprazolam pharmacokinetics in women on low-dose oral contraceptives. *J Clin Pharmacol* **28:**454-457.

Schuttler J and Stoeckel H (1982) [Clinical pharmacokinetics of alfentanyl (author's transl)]. *Anaesthesist* **31:**10-14.

Schwagmeier R, Alincic S and Striebel HW (1998) Midazolam pharmacokinetics following intravenous and buccal administration. *Br J Clin Pharmacol* **46:**203-206.

Scott JC and Stanski DR (1987) Decreased fentanyl and alfentanil dose requirements with age. A simultaneous pharmacokinetic and pharmacodynamic evaluation. *J Pharmacol Exp Ther* **240:**159-166.

Shou M, Hayashi M, Pan Y, Xu Y, Morrissey K, Xu L and Skiles GL (2008) Modeling, prediction, and in vitro in vivo correlation of CYP3A4 induction. *Drug Metab Dispos* **36:**2355-2370.

Smith RB, Kroboth PD, Vanderlugt JT, Phillips JP and Juhl RP (1984) Pharmacokinetics and pharmacodynamics of alprazolam after oral and IV administration. *Psychopharmacology (Berl)* **84:**452-456.

Soars MG, Grime K and Riley RJ (2006) Comparative analysis of substrate and inhibitor interactions with CYP3A4 and CYP3A5. *Xenobiotica* **36:**287-299.

Spina E, Tomson T, Svensson JO, Faigle JW and Bertilsson L (1988) Single-dose kinetics of an enteric-coated formulation of carbamazepine-10,11-epoxide, an active metabolite of carbamazepine. *Ther Drug Monit* **10:**382-385.

Staines AG, Coughtrie MW and Burchell B (2004) N-glucuronidation of carbamazepine in human tissues is mediated by UGT2B7. *J Pharmacol Exp Ther* **311:**1131-1137.

Stoehr GP, Kroboth PD, Juhl RP, Wender DB, Phillips JP and Smith RB (1984) Effect of oral contraceptives on triazolam, temazepam, alprazolam, and lorazepam kinetics. *Clin Pharmacol Ther* **36:**683-690.

Taillardat-Bertschinger A, Martinet CA, Carrupt PA, Reist M, Caron G, Fruttero R and Testa B (2002) Molecular factors influencing retention on immobilized artifical membranes (IAM) compared to partitioning in liposomes and n-octanol. *Pharm Res* **19:**729-737.

Tassaneeyakul W, Veronese ME, Birkett DJ, Doecke CJ, McManus ME, Sansom LN and Miners JO (1992) Co-regulation of phenytoin and tolbutamide metabolism in humans. *Br J Clin Pharmacol* **34:**494-498.

Tchaparian E, Tang L, Xu G, Huang T and Jin L (2008) Cell Based Experimental Models as Tools for the Prediction of Human Intestinal Absorption, in: *15th North American ISSX Meeting* San Diego.

Thummel KE, O'Shea D, Paine MF, Shen DD, Kunze KL, Perkins JD and Wilkinson GR (1996) Oral first-pass elimination of midazolam involves both gastrointestinal and hepatic CYP3A-mediated metabolism. *Clin Pharmacol Ther* **59:**491-502.

Tomson T, Tybring G and Bertilsson L (1983) Single-dose kinetics and metabolism of carbamazepine-10,11-epoxide. *Clin Pharmacol Ther* **33:**58-65.

Tran JQ, Kovacs SJ, McIntosh TS, Davis HM and Martin DE (1999) Morning spot and 24-hour urinary 6 beta-hydroxycortisol to cortisol ratios: intraindividual variability and correlation under basal conditions and conditions of CYP 3A4 induction. *J Clin Pharmacol* **39:**487-494.

Varma MV, Gardner I, Steyn SJ, Nkansah P, Rotter CJ, Whitney-Pickett C, Zhang H, Di L, Cram M, Fenner KS and El-Kattan AF (2012) pH-Dependent solubility and permeability criteria for provisional biopharmaceutics classification (BCS and BDDCS) in early drug discovery. *Mol Pharm* **9:**1199-1212.

Vickers S, Duncan CA, Chen IW, Rosegay A and Duggan DE (1990) Metabolic disposition studies on simvastatin, a cholesterol-lowering prodrug. *Drug Metab Dispos* **18:**138-145.

Volgyi G, Deak K, Vamos J, Valko K and Takacs-Novak K (2008) RPTLC determination of log P of structurally diverse neutral compounds. *JPlanar Chromatogr* **21:**143-149.

Von Moltke LL, Greenblatt DJ, Granda BW, Duan SX, Grassi JM, Venkatakrishnan K, Harmatz JS and Shader RI (1999) Zolpidem metabolism in vitro: responsible cytochromes, chemical inhibitors, and in vivo correlations. *Br J Clin Pharmacol* **48:**89-97.

von Richter O, Glavinas H, Krajcsi P, Liehner S, Siewert B and Zech K (2009) A novel screening strategy to identify ABCB1 substrates and inhibitors. *Naunyn Schmiedebergs Arch Pharmacol* **379:**11-26.

Vozeh S, Uematsu T, Guentert TW, Ha HR and Follath F (1985) Kinetics and electrocardiographic changes after oral 3-OH-quinidine in healthy subjects. *Clin Pharmacol Ther* **37:**575-581.

Wallin A and Herngren L (1985) Distribution of phenobarbital in whole blood during pregnancy and perinatally--an in vitro study. *Eur J Clin Pharmacol* **29:**187-191.

Walsky RL and Obach RS (2004) Validated assays for human cytochrome P450 activities. *Drug Metab Dispos* **32:**647-660.

Wan H, Ahman M and Holmen AG (2009) Relationship between brain tissue partitioning and microemulsion retention factors of CNS drugs. *J Med Chem* **52:**1693-1700.

Wandel C, Witte JS, Hall JM, Stein CM, Wood AJ and Wilkinson GR (2000) CYP3A activity in African American and European American men: population differences and functional effect of the CYP3A4*1B5'-promoter region polymorphism. *Clin Pharmacol Ther* **68:**82-91.

Weaver R, Graham KS, Beattie IG and Riley RJ (2003) Cytochrome P450 inhibition using recombinant proteins and mass spectrometry/multiple reaction monitoring technology in a cassette incubation. *Drug Metab Dispos* **31:**955-966.

Wilensky AJ, Friel PN, Levy RH, Comfort CP and Kaluzny SP (1982) Kinetics of phenobarbital in normal subjects and epileptic patients. *Eur J Clin Pharmacol* **23:**87-92.

Williams JA, Ring BJ, Cantrell VE, Jones DR, Eckstein J, Ruterbories K, Hamman MA, Hall SD and Wrighton SA (2002) Comparative metabolic capabilities of CYP3A4, CYP3A5, and CYP3A7. *Drug Metab Dispos* **30:**883-891.

Winiwarter S, Bonham NM, Ax F, Hallberg A, Lennernas H and Karlen A (1998) Correlation of human jejunal permeability (in vivo) of drugs with experimentally and theoretically derived parameters. A multivariate data analysis approach. *J Med Chem* **41:**4939-4949.

Wong KS, Kenseth J and Strasburg R (2004) Validation and long-term assessment of an approach for the high throughput determination of lipophilicity (log POW) values using multiplexed, absorbance-based capillary electrophoresis. *J Pharm Sci* **93:**916-931.

Wong SL, Locke C, Staser J and Granneman GR (1998) Lack of multiple dosing effect of sertindole on the pharmacokinetics of alprazolam in healthy volunteers. *Psychopharmacology (Berl)* **135:**236-241.

Wong YY, Ludden TM and Bell RD (1983) Effect of erythromycin on carbamazepine kinetics. *Clin Pharmacol Ther* **33:**460-464.

Woo E and Greenblatt DJ (1979) Pharmacokinetic and clinical implications of quinidine protein binding. *J Pharm Sci* **68:**466-470.

Xu Y, Zhou Y, Hayashi M, Shou M and Skiles GL (2011) Simulation of clinical drug-drug interactions from hepatocyte CYP3A4 induction data and its potential utility in trial designs. *Drug Metab Dispos* **39:**1139-1148.

Zhu C, Jiang L, Chen TM and Hwang KK (2002) A comparative study of artificial membrane permeability assay for high throughput profiling of drug absorption potential. *Eur J Med Chem* **37:**399-407.
